# Lactic acid bacteria that activate immune gene expression in *Caenorhabditis elegans* can antagonise *Campylobacter jejuni* infection in nematodes, chickens and mice

**DOI:** 10.1186/s12866-021-02226-x

**Published:** 2021-06-05

**Authors:** Xing Jin, Yufeng He, Yonghua Zhou, Xiaohua Chen, Yuan-kun Lee, Jianxin Zhao, Hao Zhang, Wei Chen, Gang Wang

**Affiliations:** 1 State Key Laboratory of Food Science and Technology, Jiangnan University, Wuxi, 214122 P. R. China; 2 School of Food Science and Technology, Jiangnan University, Wuxi, 214122 P. R. China; 3 Key Laboratory of National Health and Family Planning Commission on Parasitic Disease Control and Prevention, Jiangsu Provincial Key Laboratory on Parasite and Vector Control Technology, Jiangsu Institute of Parasitic Diseases, Wuxi, 214064 P. R. China; 4 College of Life Sciences and Environment, Hengyang Normal University, Hengyang, 421008 P. R. China; 5 Department of Microbiology & Immunology, National University of Singapore, Singapore, 117597 Singapore; 6 International Joint Research Laboratory for Probiotics, Jiangnan University, Wuxi, 214122 P. R. China; 7 (Yangzhou) Institute of Food Biotechnology, Jiangnan University, Yangzhou, 225004 P. R. China; 8 National Engineering Research Center for Functional Food, Jiangnan University, Wuxi, 214122 P. R. China; 9 Wuxi Translational Medicine Research Center and Jiangsu Translational Medicine Research Institute Wuxi Branch, Wuxi, 214122 P. R. China; 10 Beijing Innovation Centre of Food Nutrition and Human Health, Beijing Technology and Business University (BTBU), Beijing, 100048 P. R. China

**Keywords:** Lactic acid bacteria, *Campylobacter jejuni*, *Caenorhabditis elegans*, Life-span, Immune gene

## Abstract

**Background:**

*Campylobacter jejuni* is the major micro-bacillary pathogen responsible for human coloenteritis. Lactic acid bacteria (LAB) have been shown to protect against *Campylobacter* infection. However, LAB with a good ability to inhibit the growth of *C. jejuni* in vitro are less effective in animals and animal models, and have the disadvantages of high cost, a long cycle, cumbersome operation and insignificant immune response indicators. *Caenorhabditis elegans* is increasingly used to screen probiotics for their anti-pathogenic properties. However, no research on the use of *C. elegans* to screen for probiotic candidates antagonistic to *C. jejuni* has been conducted to date.

**Results:**

This study established a lifespan model of *C. elegans*, enabling the preselection of LAB to counter *C. jejuni* infection. A potential protective mechanism of LAB was identified. Some distinct LAB species offered a high level of protection to *C. elegans* against *C. jejuni*. The LAB strains with a high protection rate reduced the load of *C. jejuni* in *C. elegans*. The transcription of antibacterial peptide genes, MAPK and Daf-16 signalling pathway-related genes was elevated using the LAB isolates with a high protection rate. The reliability of the lifespan model of *C. elegans* was verified using mice and chickens infected with *C. jejuni.*

**Conclusions:**

The results showed that different LAB had different abilities to protect *C. elegans* against *C. jejuni*. *C. elegans* provides a reliable model for researchers to screen for LAB that are antagonistic to *C. jejuni* on a large scale.

**Supplementary Information:**

The online version contains supplementary material available at 10.1186/s12866-021-02226-x.

## Background

Infection by pathogenic *Campylobacter* may result in symptoms such as bloody diarrhoea, abdominal pain and fever. In many countries, *Campylobacter* species is the major microbacillary pathogen responsible for human coloenteritis [1]. In under developed countries, diarrhoeic disease is 10 times more likely to result from *Campylobacter* infection than from infection with *Escherichia coli* O157: H, *Shigella* species or *Salmonella* species [2, 3]. Some peripheral neuropathies, such as Guillain-Barré syndrome and Miller Fieher syndrome, are long-term consequences of *Campylobacter* infection [2]. *Campylobacter jejuni* (*C. jejuni*) is also one of the primary reasons for microbacillary food-borne disease in some developed countries [3]. To date, all therapies for *Campylobacter* infection involved antibiotics, especially in poultry industry. There is an urgency to develop alternative approaches due to a gradual increase in antibiotic-resistant *Campylobacter* [4]. It is also necessary to illustrate the mechanisms underlying the functions of such alternatives to support their development and implementation.

Research has increasingly shown that lactic acid bacteria (LAB) colonise the human gastrointestinal tract and play a vital role in maintaining intestinal function and well being of the host. Among the beneficial effects of LAB are bacteriostatic activities targeting pathogens such as *Escherichia coli* (*E. coli*)*, Salmonella* and *Listeria monocytogenes* [5, 6]. LAB have been shown to protect against *Campylobacter* infection. Nishiyama reported that LAB inhibited *Campylobacter* and colonisation of this pathogen was reduced by *Lactobacillus gasseri* SBT2055 isolated from healthy chicken [7]. Another study reported a decrease in *C. jejuni* invasion in the gut of turkey poults after treatment with *Lactobacillus salivarius NRRL* B-30514, suggesting that competitive exclusion can play a role [8]. Wagner et al. conducted a simulation experiment with immunodeficient and immunocompetent mice, they found that bifidobacteria and lactobacilli can increase colonisation resistance. Specifically, the results showed that bifidobacteria and lactobacilli can resist *C. jejuni* enteric persistence in the human gut [9]. To date, studies of antagonism against *C. jejuni* by LAB have mainly been conducted in vitro or in vivo. Unfortunately, some of the strains of LAB that showed a good ability to inhibit the growth of *C. jejuni* in vitro are less effective in antagonising *C. jejuni* in animals. Whether poultry or mice, all animal models have the disadvantages of high cost, a long cycle, cumbersome operation and insignificant immune response indicators, and do not permit rapid large-scale screening for LAB capable of effective antagonising *C. jejuni*.

The small, free-living (non-parasitic) soil worm *Caenorhabditis elegans* (*C. elegans*) has been widely used in biological studies as in vivo model, due to its short generation time, diminutive form and clear genetic background. To date, *C. elegans* has been used to study the effect of pathogenic microorganism, such as *Salmonella enterica*, *Pseudomonas aeruginosa, Enterococcus faecalis* and *Staphylococcus aureus* [10–14]. *C. elegans* is also increasingly used to screen for antimicrobials and probiotics [15]. In addition, institutional studies of the effects of LAB can be processed using *C. elegans*. However, no research on the use of *C. elegans* to screen for probiotic candidates antagonistic to *C. jejuni* has been conducted to date. Moreover, the molecular mechanisms underlying the protective mechanism of LAB have yet to be established. In this study, a *C. elegans* life-span experimental model was developed to investigate the response of nematodes to *C. jejuni* infection, enabling rapid evaluation of the protective effects of LAB and understanding of microbe-host interactions. At the same time, in this article, mice (with acute ileitis induced by *Toxoplasma gondii* (*T. gondii*) infection which abrogating the colonisation resistance) and chicken were also used to validate the *C. elegans* model individually [52].

## Results

### *C. jejuni* intake shortened the lifespan of *C. elegans*

**Fig. 1:**
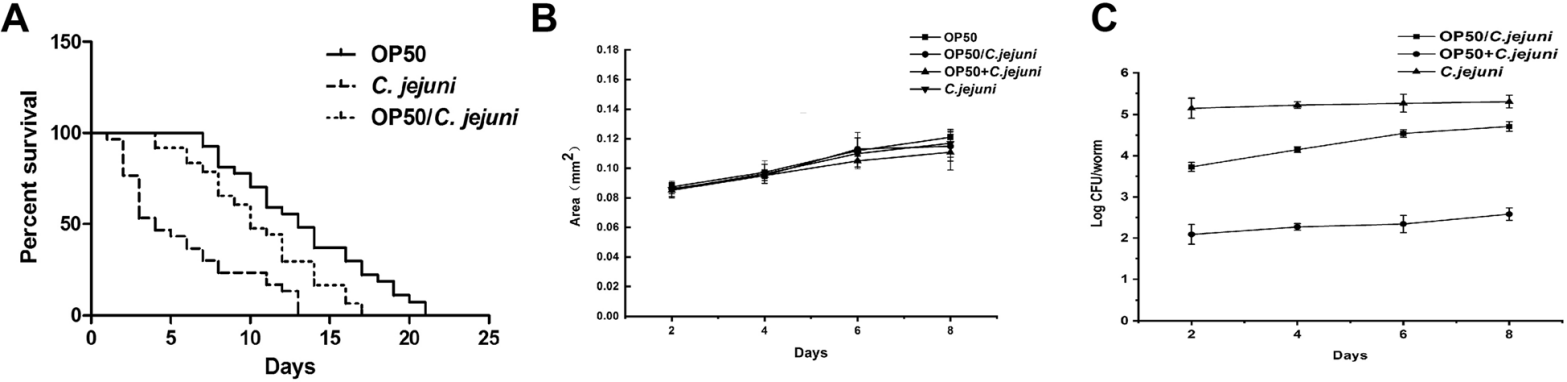
Establishment of a life-span, body size and *C. jejuni* load in *C. elegans* assay. **A** Life-span of *C. elegans* treated with *E. coli* and *C. jejuni* in different ways. Day 0 marked the arrival of the nematodes at the L4 stage before being fed thallus. **B** Body size of *C. elegans* treated with *E. coli* and *C. jejuni* in different ways. Day 0 marked the arrival of the nematodes at the L4 stage before being fed thallus. **C** *C. jejuni* load in *C. elegans* treated with *E. coli* and *C. jejuni* in different ways. Day 0 marked the point at which the nematodes were first fed *C. jejuni*

The harm caused by foodborne pathogens to the *Caenorhabditis elegans* is usually reflected in its lifespan. An experimental lifespan model of *C. elegans* was used to measure its response to *C. jejuni* infection (Fig. S1). The OP50 strain of *Escherichia coli* was used as the food source as this normally sustains nematodes on reaching the L4 stage. As shown in Fig. 1A, approximately 50% of the *C. elegans* transferred to the *C. jejuni* group died within 5 days, whereas 80% of the *C. elegans* fed with OP50 (OP50 group) were still alive after 8 days. All of the nematodes in the OP50 group died within 21 days, whereas this was 13 days for the *C. jejuni* group. When the nematodes were fed OP50 for 3 days before being treated with *C. jejuni* (OP50/*C. jejuni* group), they were more resistant to *C. jejuni* and nearly 50% were still alive on day 10. However, the lifespan of *C. elegans* was shorter in the OP50/*C. jejuni* group than in the OP50 group. In addition, the initial number of *C. jejuni* cells that were recovered from the nematodes in the OP50/*C. jejuni* group was lower than that recovered from the nematodes in the *C. jejuni* group (Fig. 1C). This may be due to the protective effect of OP50 and/or shorter time of *C. jejuni* intake. However, to allow available time for lactic acid bacteria (LAB) intervention, *C. elegans* at day 3 after the L4 stage was chosen for the lifespan assay. Although the OP50/*C. jejuni* group was less effective in infecting *C. elegans* with *C. jejuni* than the *C. jejuni* group, the lifespan of *C. elegans* was still considerably shortened compared with the OP50 group.

The body size of *C. elegans* directly reflects its growth and development, which are closely related to its energy intake. In addition to directly causing disease, pathogens may also affect the lifespan of *C. elegans* due to their inability to be metabolised by this nematode. To determine whether the lifespan of *C. elegans* was shortened due to the pathogenicity of *C. jejuni* or reduced caloric intake, the body size of *C. elegans* fed with *C. jejuni* was compared with that of *C. elegans* fed with OP50 (Fig. 1B). The nematodes in the OP50 + *C. jejuni* group were fed equal amounts of OP50 and *C. jejuni* concurrently at the L4 stage. For 8 days, the nematodes in the OP50 + *C. jejuni* and OP50/*C. jejuni* groups were nearly the same size as those in the OP50 group, which indicated that the three groups had almost the same caloric intake during this period. Although the intestinal load of *C. jejuni* in the nematodes in the OP50/*C. jejuni* group was much larger than that in the OP50 + *C. jejuni* group, the load of *C. jejuni* in both groups remained stable (Fig. 1C), indicating that the nematodes showed no preference for ingesting *C. jejuni* over *E. coli*. Therefore, substituting *C. jejuni* for *E. coli* did not lead to fasting in *C. elegans*, which would have affected its lifespan.

### LAB strains prolonged the lifespan of *C. elegans* infected by *C. jejuni*

**Fig. 2:**
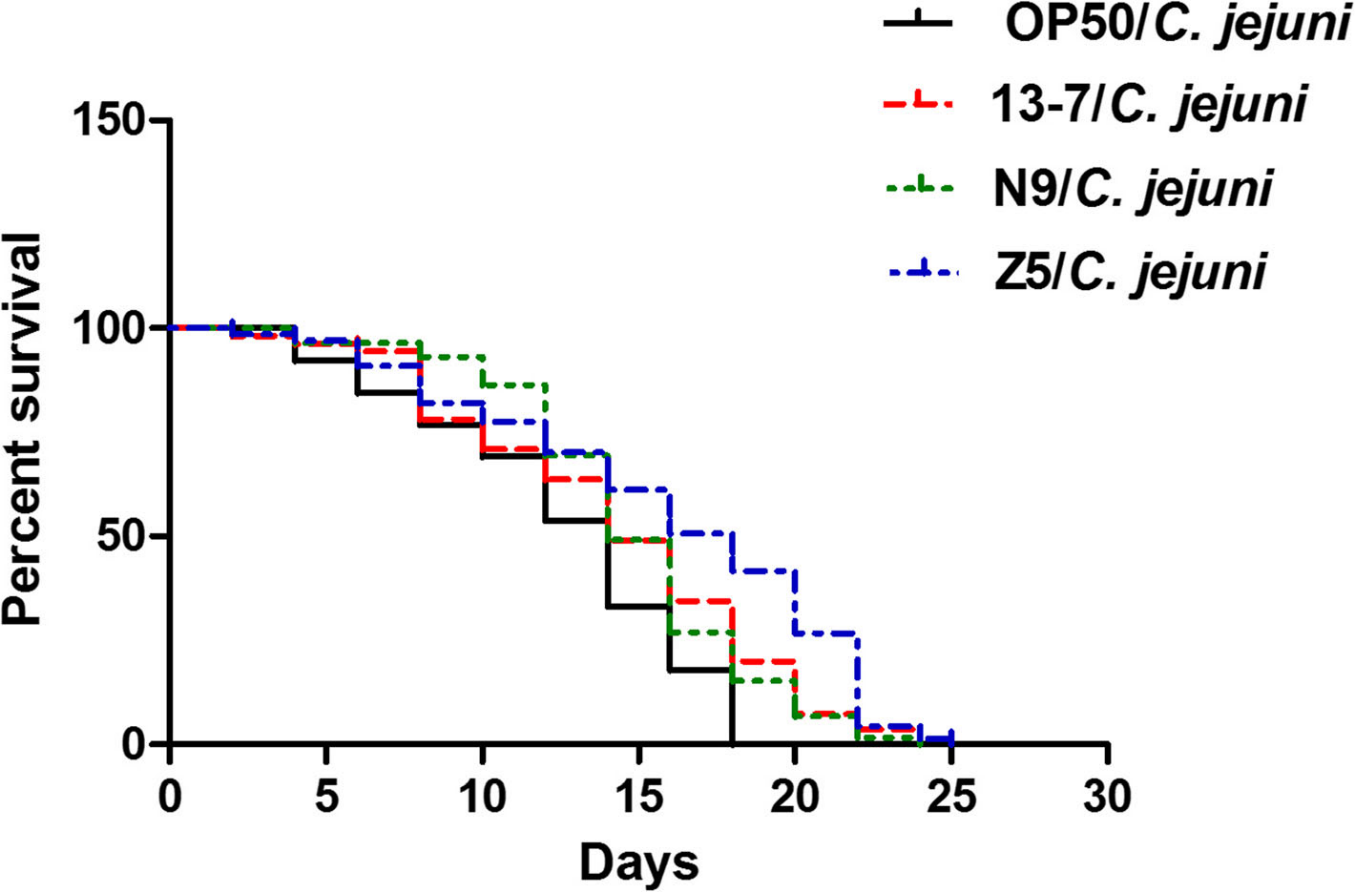
Differential effects of LAB on the survival of *C. elegans* infected with *C. jejuni*. OP50/*C. jejuni*: treated with *E. coli* OP50 for the first 3 days and then with *C. jejuni* after the L4 stage. LAB/*C. jejuni*: treated with LAB for the first 3 days and then with *C. jejuni* after the L4 stage. Day 0 marked the arrival of the nematodes at the L4 stage before being fed thallus

The lifespan of *C. elegans* after bacterial infection is a common indicator of this nematode’s antibacterial ability. To enable the rapid evaluation of the defensive effects of LAB, 44 LAB were assessed for their ability to protect *C. elegans* from *C. jejuni* infection-mediated death. On day 13, the *C. elegans* that were only fed OP50 displayed a 50% survival rate (DT50), whereas the DT50 of the *C. elegans* that were only fed *C. jejuni* was only 20%. As shown in Table 1, the LAB isolates varied in their ability to protect the live nematodes, with survival rates ranging from 15 to 60%. Among the tested isolates, some LAB provided high levels of protection, especially 13–7, Z5 and N9 (each leading to a survival rate of > 40%), and the lifespans of the nematodes in these three groups were significantly longer than the lifespan of the nematodes the OP50/*C. jejuni* group (Fig. 2).

**Table 1:** Statistical analysis of the protection effects of LAB stains on *C. elegans* infected by *C. jejuni* NCTC 11168^a^

Groups^b^	Survival (%)	DT50^c^(day)	*P*	Groups^b^	Survival (%)	DT50^c^(day)	*P*
OP50/*C. jejuni*	20.16	6.83		ZX6/*C. jejuni*	26.03	7.27	0.21
OP50	55.78	14.90	< 0.01	PC-T7/*C. jejuni*	25.56	8.85	0.21
422/*C. jejuni*	15.09	6.06	0.92	JS-WX-9-1/*C. jejuni*	29.01	9.58	0.20
B/*C. jejuni*	18.02	7.38	0.88	N8/*C. jejuni*	28.67	8.09	0.18
G14/*C. jejuni*	20.01	7.31	0.87	11,657/*C. jejuni*	31.20	10.45	0.16
X14/*C. jejuni*	16.42	7.23	0.88	YM-1/*C. jejuni*	31.29	11.13	0.11
LSQ3/*C. jejuni*	19.03	7.06	0.87	676/*C. jejuni*	27.05	10.66	0.10
591/*C. jejuni*	20.66	8.03	0.73	JS-SZ-1-5/*C. jejuni*	32.08	11.84	0.07
H17/*C. jejuni*	20.32	6.79	0.58	NCFM/*C. jejuni*	36.89	12.49	< 0.01
LGG/*C. jejuni*	21.05	7.65	0.57	N34/*C. jejuni*	37.52	12.39	< 0.01
1101/*C. jejuni*	21.03	7.55	0.57	427/*C. jejuni*	38.02	13.06	< 0.01
N29/*C. jejuni*	23.79	9.05	0.56	X13/*C. jejuni*	38.78	13.04	< 0.01
408/*C. jejuni*	21.43	8.41	0.55	720/*C. jejuni*	39.11	12.21	< 0.01
H29M–8 M/*C. jejuni*	22.17	7.81	0.55	2009/*C. jejuni*	39.48	12.16	< 0.01
730/*C. jejuni*	21.89	6.91	0.53	H33M-1/*C. jejuni*	40.12	13.09	< 0.01
H9/*C. jejuni*	22.81	8.45	0.51	13 M2/*C. jejuni*	42.01	13.56	< 0.01
ZX7/*C. jejuni*	22.95	9.18	0.46	L103/*C. jejuni*	44.41	12.67	< 0.01
430/*C. jejuni*	24.09	9.79	0.42	G20/*C. jejuni*	45.15	13.96	< 0.01
675/*C. jejuni*	22.67	7.98	0.41	1132/*C. jejuni*	45.18	13.76	< 0.01
Z7/*C. jejuni*	22.14	7.46	0.29	H27-1 L/*C. jejuni*	45.81	14.90	< 0.01
Z6/*C. jejuni*	15.03	8.32	0.27	**13–7/*C. jejuni***	47.09	14.01	< 0.01
rui/*C. jejuni*	25.81	10.46	0.26	**Z5/*C. jejuni***	57.42	15.11	< 0.01
9–5/*C. jejuni*	29.58	9.21	0.25	**N9/*C. jejuni***	43.28	13.70	< 0.01

^a^Summary of two or more separate experiments. Survival of worms on the last day (day13) of the assays was estimated with the Kaplan-Meier survival analysis

^b^OP50/*C. jejuni*: treatment with *E. coli* OP50 in the first 3 days and then with *C.jejuni* when *C. elegans* after L4 stage. OP50: treatment with *E. coli* OP50 during the whole experiment when *C. elegans* after L4 stage. LAB/ *C. jejuni*: treatment with LAB in the first 3 days and then with *C.jejuni* when *C. elegans* after L4 stage. The time of L4 stage nematodes before fed any thallus was considered 0 day

^c^DT50, the time at which half of the worms were dead

### LAB strains decreased the *C. jejuni* load in the intestine of *C. elegans*

**Fig. 3:**
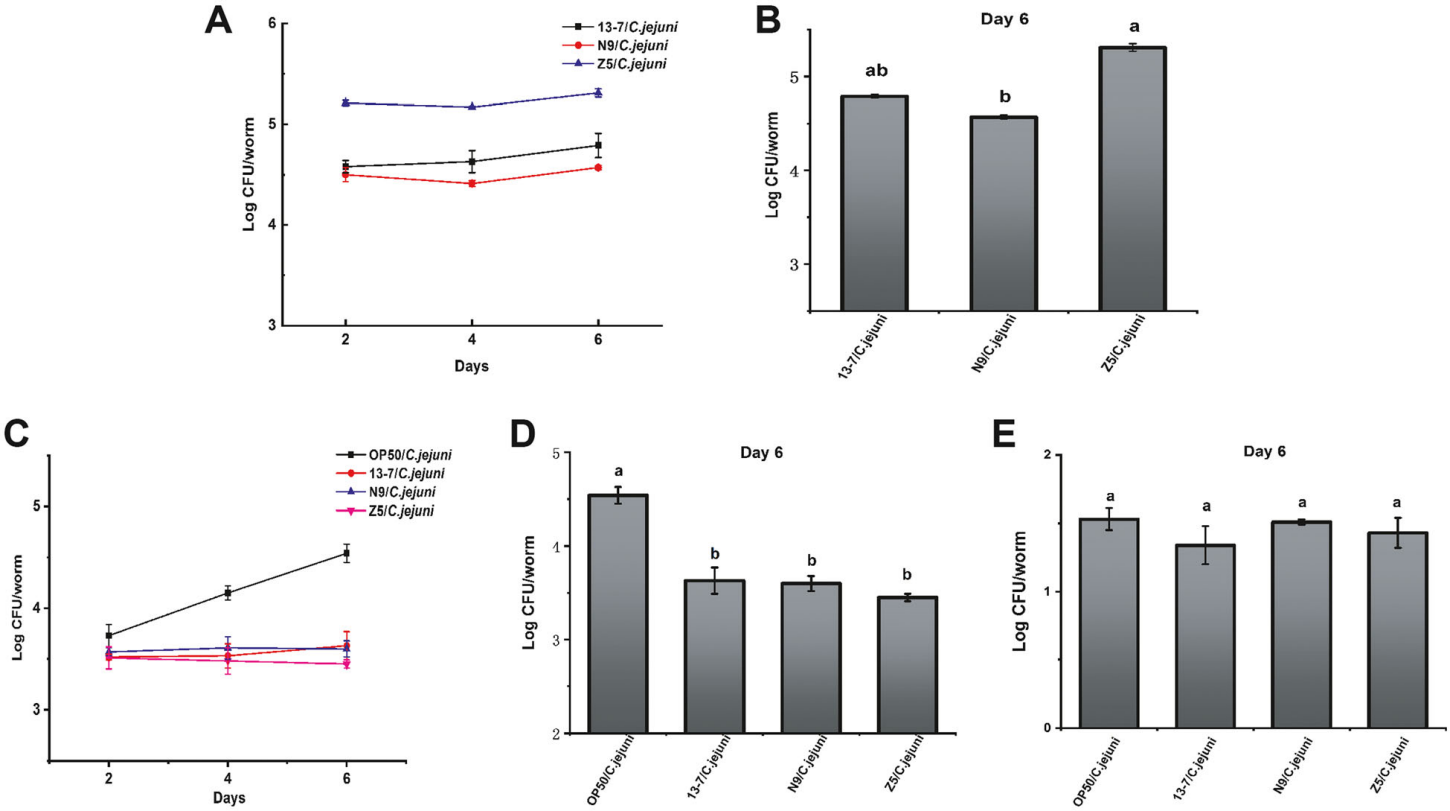
LAB, *C. jejuni* and OP50 load in the intestine of *C. elegans*. **A** and **B** LAB load. Day 0 marked the arrival of the nematodes at the L4 stage before being fed thallus. **C** and **D** *C. jejuni* load. Day 0 marked the point at which the nematodes were first fed *C. jejuni.* **E** OP50 load. Day 0 marked the arrival of the nematodes at the L4 stage before being fed thallus. The graphs show means ± SDs. Column labelled with different superscript letters (a, b) showed significant differences (*p* < 0.05). Any two columns with same superscript letter

Diminishing pathogenic bacterial colonisation in the intestinal tract is probably one of the underlying mechanisms of LAB in prolonging the lifespan of nematodes. In addition, LAB cells colonising the intestine of *C. elegans* play a role in decreasing the number of pathogenic bacteria. To investigate whether an association exists between the lifespan of *C. elegans* and the loadings of different bacteria in their intestines, the numbers of LAB, *C. jejuni* and *E. coli* OP50 in the intestines of *C. elegans* were counted. The 44 LAB strains that showed various levels of protection were evaluated for their ability to persist in the intestines of *C. elegans* from day 2 to day 6. The loads of LAB on 13–7, Z5 and N9, which showed a strong ability to protect *C. elegans* against death, exceeded log 10^4.5^ colony-forming unit (CFU)/nematode during the assay (Fig. 3A and B). In contrast, the groups showed low levels of protection for the nematodes in the lifespan assay, and had the lowest colonisation levels of LAB (Table S2). The *C. jejuni* loads in *C. elegans* from day 2 to day 6 were also determined. As shown in Fig. 3C and D, the *C. jejuni* loads in *C. elegans* treated with 13–7, Z5 and N9 were almost 1.5 orders of magnitude lower than that in the OP50/*C. jejuni* group (log 10^4.5^ CFU/nematode). In contrast, the groups offering low levels of protection in terms of lifespan did not significantly decrease the *C. jejuni* load (Table S3). There were no obvious differences in the OP50 load between the OP50/*C. jejuni* group and the other LAB intervention groups (log 10^1.2^–10^1.5^ CFU/nematode) on day 6 (Fig. 3E and Table S4). The results of a correlation analysis showed that the LAB and *C. jejuni* loads were highly positively correlated and highly negatively correlated with the survival of *C. elegans*, respectively. In addition, the OP50 load was not correlated with the survival of *C. elegans* (Table 2).

**Table 2:** Correlation analysis of LAB colonization and *C.jejuni* load in the intestine and survival of *C. elegans*

Comparison Groups		Pearson Correlation
Colonization of LAB	Survival of *C. elegans*	R ^2^ = 0.517^**^
*C. jejuni* load	Survival of *C. elegans*	R^2^ = − 0.615^**^
Colonization of LAB	*C. jejuni* load	R ^2^ = − 0.424*
OP50 load	Survival of *C. elegans*	R^2^ = − 0.013

* Indicates statistically significant differences at *p* < 0.05

** Indicates statistically significant differences at *p* < 0.01

### No change on body size and pharyngeal pumping of *C. elegans* infected by *C. jejuni* under LAB intervention

**Fig. 4:**
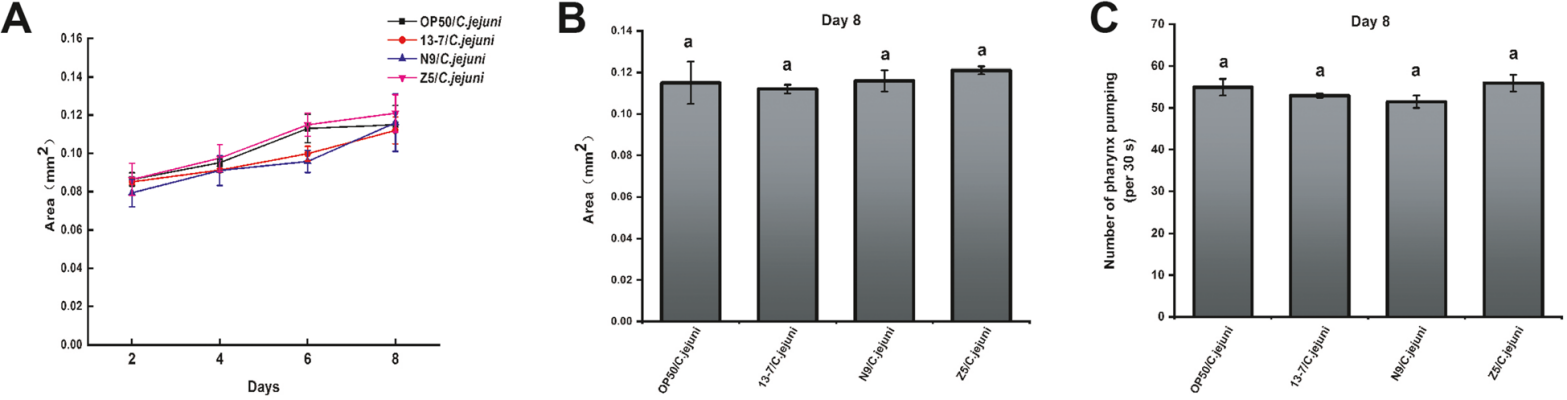
Effects of LAB on body size and pharynx pumping of *C. elegans*. **A** and **B** Body size (area) of *C. elegans* treated with LAB and *C. jejuni* on day 8. Day 0 marked the arrival of the nematodes at the L4 stage before being fed thallus. **C** Effects of LAB on the pharynx pumping of *C. elegans* infected by *C. jejuni*. Pharynx pumping (per 30s) of *C. elegans* treated with LAB and *C. jejuni* on day 8. All day 0 marked the arrival of the nematodes at the L4 stage before being fed thallus. The graphs show means ± SDs. Column labelled with different superscript letters (a, b) showed significant differences (*p* < 0.05). Any two columns with same superscript letter

As mentioned, the body size of *C. elegans* directly reflects its growth and development, which are closely related to its energy intake. In addition to body size, pharyngeal pumping represents the feeding capacity of *C. elegans* and is another key index for physiological activities. To determine whether the longevity effects of LAB were the result of caloric reduction, the body size (Table S5) and pharyngeal pumping rate (Table S6) of *C. elegans* fed with 44 LAB strains and *C. jejuni* (LAB/*C. jejuni* groups) were compared with those of the nematodes in the OP50/*C. jejuni* group. The nematodes in the OP50/*C. jejuni* group and those in the 13–7, Z5 and N9 intervention groups showed little difference in body size (Fig. 4A and B). Similarly, little difference was observed in pharyngeal pumping between the groups. Figure 4C showed that the pharyngeal pumping rate ranged from 50 to 58 per 30s in the OP50/*C. jejuni* group and 13–7, Z5 and N9 intervention groups. There were no significant differences in body size or pharyngeal pumping of nematodes treated by these LAB, thus demonstrating the varied longevity of *C. elegans* treated with *C. jejuni*.

### Influence on *C. jejuni* growth by co-culturing with *E. coli* OP50

**Fig. 5:**
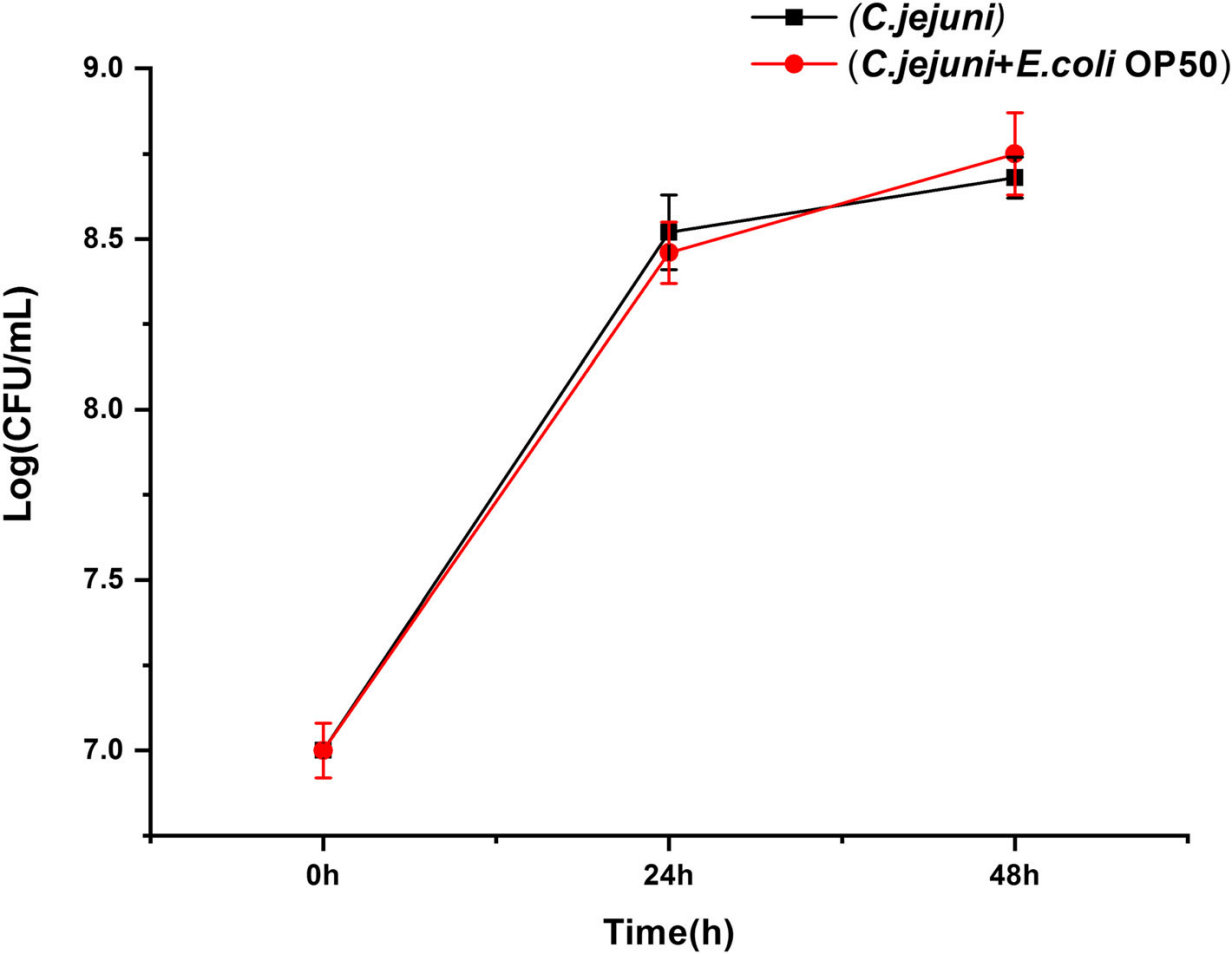
Live cell counts of *C. jejuni* in the co-culture with *E.coli* OP50 strain

To investigate whether the substances produced by *E. coli* OP50, such as certain bacteriocins, could kill *C. jejuni*, the viability of *C. jejuni* cultured with and without live *E. coli* OP50 was measured. As shown in Fig. 5, the number of *C. jejuni* increased in both tests after 24 h of incubation and did not exhibit a significant difference between the tests. Furthermore, the number of *C. jejuni* was uninfluenced after the live *E. coli* OP50 were added to the growing *C. jejuni* after 48 h of incubation.

### LAB strains upregulated the immune gene transcription

**Fig. 6:**
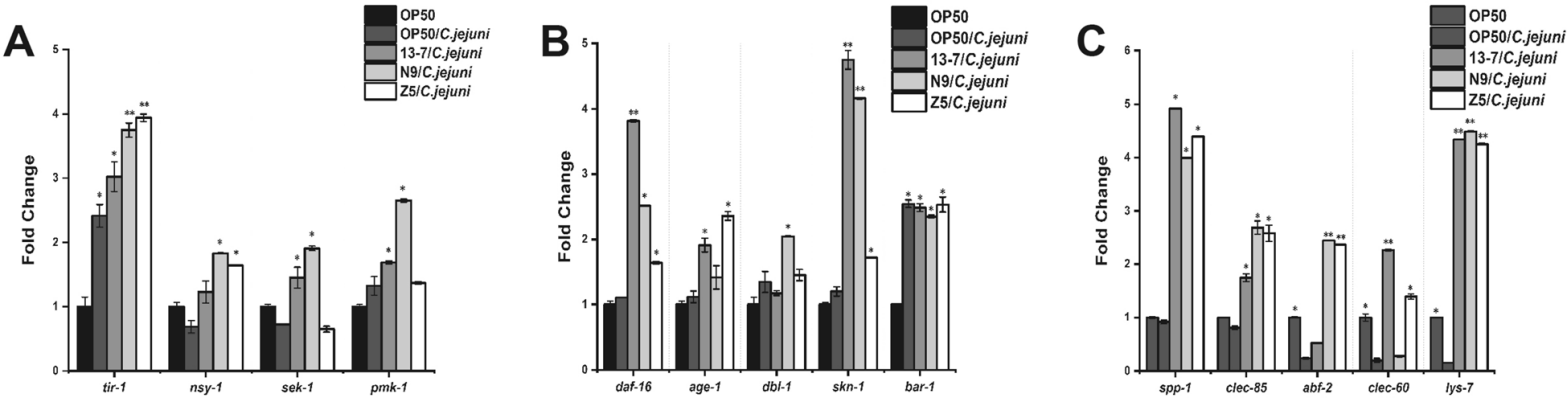
Differential effects of LAB on transcription of immune genes of *C. elegans* on day 6. Day 0 marked the arrival of the nematodes at the L4 stage before being fed thallus. * Indicates statistically significant differences at *p* < 0.05. ** Indicates statistically significant differences at *p* < 0.01

Regulation of the host’s immune system through pivotal signalling pathways is an underlying mechanism by which LAB can prolong the lifespan of a nematode and protect it against pathogen infection. To determine the mechanism by which LAB protected *C. elegans* against *C. jejuni* infection, the transcription levels of 14 immune genes (*tir-1*, *nsy-1*, *sek-1*, *pmk-1*, *spp-1*, *clec-85*, *abf-2*, *clec-60*, *lys-7*, *daf-16*, *age-1*, *dbl-1*, *skn-1* and *bar-1*) of *C. elegans* on day 6 were compared between the OP50, OP50/*C. jejuni*, and LAB/*C. jejuni* groups. As shown in Fig. 6A, B and C, the transcription of *tir-1* and *pmk-1* (MAPK signalling pathway genes) and *bar-1* (an antioxidant gene) increased to some extent when *C. elegans* was infected with *C. jejuni*. In addition, slight increases were observed in some of the defence immune genes of *C. elegans* infected with *C. jejuni*, such as *daf-16* and *age-1* (Daf-16 signalling pathway genes) and *dbl-1* (a TGF-β signalling pathway gene).

The 14 immune genes of *C. elegans* were also examined after treatment with 44 LAB strains, which revealed a variation in the protection against *C. jejuni*-induced nematode mortality (Table S7). It was found that the expression level of the immune genes of *C. elegans* did not change significantly after LAB intervention without *C. jejuni* infection, indicating that the LAB strains were safe for a healthy host (Table S8). As shown in Fig. 6A, B and C, the treatments of nematodes with 13–7, N9 and Z5 significantly enhanced the transcription of the MAPK signalling pathway genes *nsy-1*, *sek-1* and *pmk-1*, the antioxidant gene *skn-1*, and the Daf-16 signalling pathway genes *age-1* and *daf-16*. The transcription levels of the antibacterial peptide genes *spp-1*, *clec-85* and *lys-7* in these LAB intervention groups were 3–4 times higher than those in the group infected with *C. jejuni* alone. These data suggest that the ability of certain LAB to protect nematodes against *C. jejuni*-induced mortality was correlated with their influence on the transcription levels of immune genes. In contrast, when *C. elegans* was treated with LAB offering low levels of protection for survival, the transcription levels of their defence genes were almost identical to those of *C. elegans* infected with *C. jejuni* alone (Table S7).

### LAB strains that prolonged the lifespan of *C. elegans* decreased the *C. jejuni* load in mice with *T. gondii*-induced acute ileitis

**Fig. 7:**
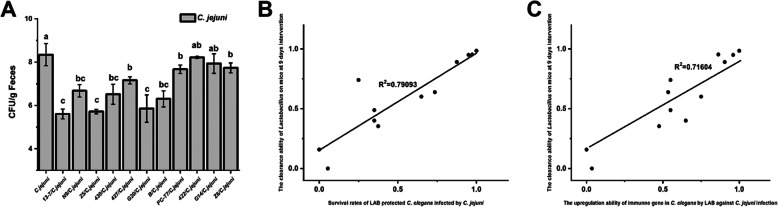
Differential effects of LAB on *C. jejuni* load in mice infected by *C. jejuni* and correlation analysis between *C. elegans* and mice models. **A** Culturable *C. jejuni* in mice faeces on day 9. The graphs show means ± SDs. Column labelled with different superscript letters (a, b, c) showed significant differences (*p* < 0.05). Any two columns with same superscript letter. **B** Correlation analysis of ability of LAB strains to clear *C. jejuni* in infected mice on day 5 and the survival rate of *C. jejuni*-infected nematodes under LAB treatment. **C** Correlation analysis of ability of LAB strains to clear *C. jejuni* in infected mice on day 9 and upregulate immune gene transcription to protect *C. elegans* against *C. jejuni* infection

The LAB that protect nematodes against bacterial infection might be inapplicable to mammals due to the obvious differences between the organisms. To investigate whether the LAB screened from *C. elegans* had the same effects in mammals, 11 LAB strains (including 13–7, Z5 and N9) with different effects on the immune gene transcription of *C. elegans* were further investigated to determine their abilities to decrease the *C. jejuni* load in mice. *Toxoplasma gondii* was used to induce acute ileitis to avoid colonisation resistance in normal mice. Seven days after *T. gondii* infection, the mice developed acute ileitis and most died; thus, the *C. jejuni* loads were determined on day 9. The *C. jejuni* load in the faeces of mice in the *C. jejuni*-infected group reached 10^8^ CFU/g (Fig. 7A). The LAB strains that showed the strongest ability to protect *C. elegans* (i.e. 13–7, Z5 and G20) also exerted a superior suppressive effect on the *C. jejuni* load (< 10^6^ CFU/g of faeces) in the intestinal tract of mice. Strains 422 and G14, which showed poor protective effects in terms of the lifespan of *C. elegans*, correspondingly played an inconspicuous role in suppressing the *C. jejuni* load (~ 10^8^ CFU/g of faeces). Consistently with this, PC-T7 and Z6, which had previously offered moderate and low levels of protection, respectively, to *C. elegans*, showed slight suppressive effects on the *C. jejuni* load (10^7^–10^8^ CFU/g of faeces). Meanwhile, N9 and 430, which had previously offered high and moderate levels of protection to *C. elegans*, respectively, had moderate suppressive effects on the *C. jejuni* load (10^6^–10^7^ CFU/g of faeces).

A correlation analysis was conducted to examine the relationship between the ability of the 11 LAB strains to remove *C. jejuni* in mice and the survival rate of *C. jejuni-*infected nematodes or the uprefulation ability of immune gene (*spp-1*) in *C. elegans* by LAB against *C. jejuni* infection. The correlation coefficient R^2^ reached 0.79093 and 0.71604, indicating that the *C. jejuni*-antagonistic activity of LAB in *C. elegans* by upregulating worms immune gene transcription to prolong life was significantly correlated with the *C. jejuni*-antagonistic activity of LAB in mice (Fig. 7B and C).

### LAB strains that prolonged the lifespan of *C. elegans* decreased the *C. jejuni* load in chickens

**Fig. 8:**
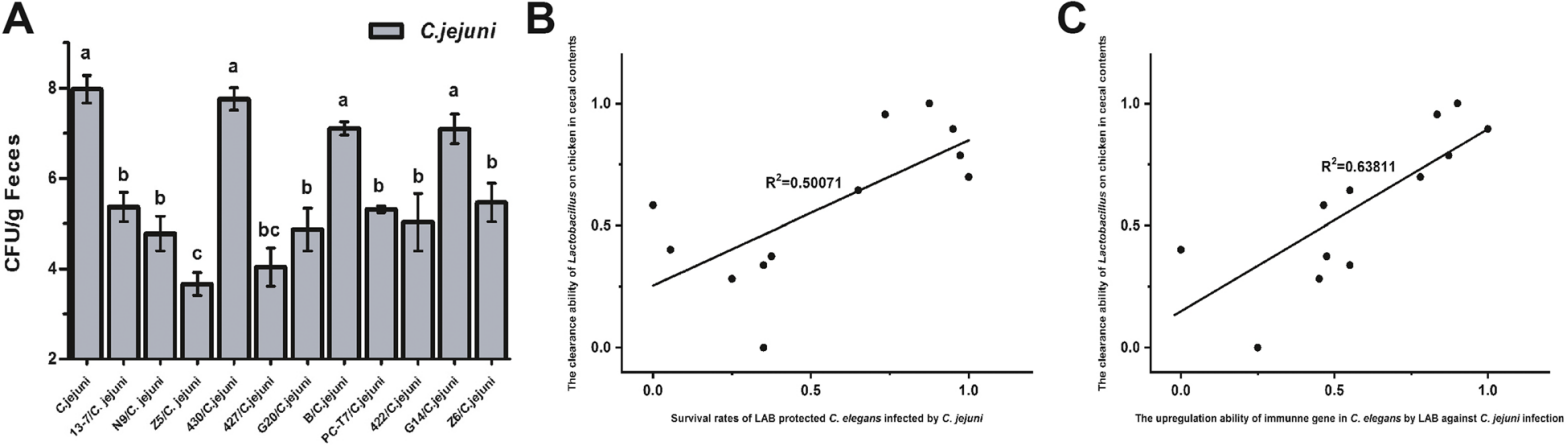
Differential effects of LAB on *C. jejuni* load in chicken infected by *C. jejuni*. **A** Culturable *C. jejuni* in chicks’ cecal contents on day 23. The graphs show means ± SDs. Column labelled with different superscript letters (a, b, c) showed significant differences (*p* < 0.05). Any two columns with same superscript letter. **B** Correlation analysis of ability of LAB strains to clear *C. jejuni* in infected chicks’ cecal contents and the survival rate of *C. jejuni*-infected nematodes under LAB treatment. **C** Correlation analysis of ability of LAB strains to clear *C. jejuni* in infected chicks’ cecal contents and upregulate immune gene transcription to protect *C. elegans* against *C. jejuni* infection

Outbreaks of campylobacteriosis can occur if humans ingest undercooked poultry contaminated with *C. jejuni*. Studies have shown that LAB applied to fodder could reduce *Campylobacter* colonisation in poultry and stop the outbreak of this disease at its source. To investigate whether the LAB screened from *C. elegans* had the same effects in poultry, 11 LAB strains with different effects on immune gene transcription in *C. elegans* were investigated to determine their abilities to remove *C. jejuni* in chickens. The inhibitory effect of LAB on *C. jejuni* colonisation in the cecum of chickens was examined. Approximately 24 h after hatching, chickens were inoculated orally with *C. jejuni*, and then LAB were administered daily for 2 weeks. The CFU count of *C. jejuni* in the cecum of chickens in all groups was evaluated on day 23. The average value of *C. jejuni* increased to 10^8^ CFU/g of cecal content in the *C. jejuni*-infected group (Fig. 8A). Strains Z5 exerted significant suppressive effect on *C. jejuni* colonisation in the cecum of chickens, which resulted in the reduction of the *C. jejuni* load to below 10^4^ CFU/g of cecal content in this group. Meanwhile, strains 430, B and G14 showed poor abilities to remove *C. jejuni* from the cecum of chickens. The *C. jejuni* loads in these groups were > 10^7^ CFU/g of the cecal content. The effects of these three strains in eliminating *C. jejuni* in *C. elegans* were similar to their effects in eliminating *C. jejuni* in chickens. However, strains 430 and B exhibited moderate *C. jejuni* antagonism in *C. elegans*, which were stronger than their performance in chickens. In addition, 13–7, N9, G20, PC-T7, 422 and Z6 showed moderate ability to eliminate *C. jejuni* in the cecal content of chickens, with a *C. jejuni* load of 10^4.8^–10^5.5^ CFU/g of faeces. Two of those strains (422 and Z6) presented a certain degree of inconsistency in the *C. jejuni* antagonism in chickens and nematodes.

A correlation analysis was conducted to examine the relationship between the ability of the 11 LAB strains to remove *C. jejuni* in chickens and the survival rate of *C. jejuni-*infected nematodes or the uprefulation ability of immune gene (*spp-1*) in *C. elegans* by LAB against *C. jejuni* infection. The R^2^ value reached 0.50071 and 0.63811, indicating that the *C. jejuni*-antagonistic activity of LAB in *C. elegans* by upregulating worms immune gene transcription to prolong life was correlated with their *C. jejuni*-antagonistic activity in chickens (Fig. 8B and C).

## Discussion

The model organism *C. elegans* has been treated with numerous pathogenic microorganisms from animals and humans to examine the relationships between pathogenic bacteria and their hosts [10–14]. In this study, a lifespan experimental model of *C. elegans* was established to examine the response of this nematode to *C. jejuni* infection. Our experiments revealed that LAB strains varied in their ability to defend *C. elegans* against infection from *C. jejuni* (NCTC 11168). In addition, the antagonistic effects of LAB isolates on *C. jejuni* in *C. elegans* were verified in both mice and chickens. The lifespan experimental model of *C. elegans* not only provided a useful method of screening for LAB candidates with the potential to mitigate *C. jejuni* infection, but also helped to explain the defence mechanism imparted by the LAB isolates. The findings also indicated that LAB defended *C. elegans* not only by inhibiting *C. jejuni* colonisation in the intestines, but also by activating the defence immune genes of *C. elegans*.

The natural physiological activities of nematodes are usually halted by the colonisation of invading pathogens [17–19]. Some LAB, such as *Lacticaseibacillus rhamnosus* [20], *Lactobacillus acidophilus* [21], *Lactobacillus fermentum* [13], *Lactobacillus gasseri* and *Lactobacillus plantarum* [22], have been reported to show high shielding efficiencies to nematodes from pathogens, to diminish the risk of bacterial colonisation in the intestinal tract, and to prolong the lifespan of nematodes. The intestinal colonisation of *C. jejuni* may occur in humans and animals, and is often observed in poultry. The binding of fimbrial adhesins to the host’s intestinal tract is a precursor to *C. jejuni* nosogenesis, which leads to diseases such as diarrhoea [23, 24]. Many studies have shown that *C. jejuni* colonisation and growth in the host’s intestinal tract can be reduced by some LAB, such as *Bifidobacterium* and *Lactobacillus salivarius* [25, 26]. The results of the present study confirmed that the level of *C. jejuni* in the intestinal tract of *C. elegans* was affected differentially by LAB strains, which offered strong to weak levels of protection. The *C. jejuni* load in *C. elegans* was strongly correlated with its lifespan, which indicated that the inhibition of *C. jejuni* colonisation in the intestinal tract was one of the mechanisms through which LAB offered protection to *C. elegans*. As reported, some foodborne pathogens, such as *Salmonella enterica* serovar Typhimurium [21] and entero-invasive *E. coli* [27], can colonise the intestinal tract of nematodes to form a persistent lethal infection. The colonised LAB in the intestine continuously produce antibacterial products, and can also occupy the adhesion sites of pathogens, which jointly exert antibacterial effects [54]. However, in this study, the LAB load and *C. jejuni* load in *C. elegans* were negatively correlated, indicating that some LAB may alleviate the damage caused by infected pathogens in other ways, such as through the regulation of immune gene expression in nematodes.

In non-mammalian taxa, mice and some primates, calorie restriction is normally used to increase longevity and ease the consequences of ageing [28–30]. Researchers have also investigated the caloric intake of *C. elegans* after LAB intervention through measurements of body size and the pharyngeal pumping rate [10–14]. But in this article, under the intervention of LAB, there was no relationship between *C. jejuni-* infected *C. elegans* lifespan extension and its morphological or pharyngeal pumping.

With the growth and reproduction of LAB in a host’s intestine, the immune system is regulated through pivotal signalling pathways with LAB involvement [31]. The host’s immune response to pathogens may be induced by LAB [32]. In addition, LAB strains with protective efficacy, such as *L. rhamnosus* GG [33], *L. plantarum* [34] and *Lactobacillus delbrueckii* [35], can enhance the host’s immune defence against *E. coli*, *S. typhimurium* and *Streptococcus pyogenes* infection by exciting the MAPK signalling pathway and through toll-like receptors. Moreover, LAB may defend the host by adjusting gene expression through cytokine and chemokine activity [36, 37]. Another possible mechanism is the enhancement of the membrane barrier [38, 39]. *Caenorhabditis elegans* has two main signalling pathways: P38-MAPK and TGF-β. Its specific defence system, including antimicrobial responses, falls short of an adaptive immune system [40]. Nevertheless, the upregulation of MAPK pathway gene (*tir-1*, *nys-1*, *sek-1* and *pmk-1*) expression can enable *C. elegans* to resist various microbial infections. These findings demonstrated the activation of the MAPK pathway through LAB intervention [31]. In the present study, the LAB strains offered good levels of protection for *C. elegans* against *C. jejuni* infection, and significantly enhanced the transcription of the MAPK pathway genes. As a downstream molecule regulated by the MAPK signalling pathway, the fork-head family transcription factor DAF-16 has been shown to adjust gene expression to improve dauer formation in the larval phase of nematodes, and to increase resistance and longevity when mature [41–43]. The results of the present study were consistent with the above findings, suggesting that the studied LAB strains prolonged the lifespan of *C. elegans* by upregulating DAF-16 via the MAPK signalling pathway.

In addition, it is considered that the cluster of C-type lectins can mask bacterial attachment and enhance resistance to microbial infection [44]. The increased levels of *clec-60*, *clec-85*, *spp-1* and *abf-2* upon LAB pre-treatment in this study also indicate the ability of some LAB to protect nematodes against *C. jejuni* infection. *Skn-1* and *bar-1* may also play critical roles in defending a host and prolonging its survival. The increased level of *skn-1* in pre-treated nematodes has been found to be due to antioxidant defence followed by their improved survival [45–47]. Partly consistent with this claim, *skn-1* transcription in the current study was significantly enhanced by the LAB strains that prolonged the lifespan of *C. elegans*. Although the TGF-β pathway is a major defence system in *C. elegans*, *dbl-1* showed almost no obvious changes in the transcription level across the LAB intervention groups, indicating that this gene may not play an essential role in protecting against *C. jejuni* infection. Moreover, the immune genes of *C. elegans* without *C. jejuni* infection did not change significantly after intervention by the LAB strains. The studied LAB only played a significant role when the nematodes received external infection, suggesting that LAB are safe for a healthy host.

As the growth temperature of *C. elegans* is much lower than the temperature in the human gut, and the microbiota of *C. elegans* is much simpler than that of a mammal, the reliability of this lifespan model of *C. elegans* in testing the efficiency of LAB antagonism against *C. jejuni* colonisation in mammals should be confirmed. In addition, because the short cycle of acute infection in mice represents a research limitation, chickens were used to investigate the long-term effects in this study. Nevertheless, the *C. jejuni-*infected chicken model has been widely used in screening probiotics for feed with *C. jejuni* antagonism, which could be applied as antibiotic substitutes [16]. The significant correlation between the ability of LAB to remove *C. jejuni* in mice and chicken and their ability to upregulate immune gene transcription to protect *C. elegans* against *C. jejuni* infection indicates that the lifespan model of *C. elegans* infected with *C. jejuni* can be applied to some extent to *C. jejuni-*infected mammals and birds*.* Although the correlation between the different models was relatively good, it was undeniable that some strains had different antibacterial effects in these models. The reason for the difference may be physiological differences (such as body temperature) on different hosts or different growth characteristics of LAB in the body (the ability of colonization or production of antibacterial substances). The results indicate that *C. elegans* is a good model organism for screening *C. jejuni-*resistive LAB strains on a large scale.

In this study, the observed effects of LAB strains on the antagonistic ability of *C. jejuni* infection in nematodes with its genome determinants were also investigated. Taking *L. plantarum* N9 as an example, a glycosyltransferase-encoding gene that differed to that of other *L. plantarum* strains was found in COG and NR annotations. This gene regulates the synthesis of extracellular polysaccharides in bacterial strains. Exopolysaccharides have been proven to have a series of physiological health care effects, such as antioxidant properties, immune regulation and resistance to pathogenic bacteria. Therefore, the analysis results suggest that the core functional genes of LAB endow them with the ability to alleviate the infection of *C. jejuni* in nematodes.

## Conclusion

This study established a lifespan model of *C. elegans* that can measure the response of this nematode to *C. jejuni*. Different LAB species exhibited different effects on the response of *C. elegans* to infection with *C. jejuni*. The inhibition of intestinal colonisation by *C. jejuni* may have been one of the mechanisms through which LAB protected *C. elegans*, and the protection offered by LAB may also have derived partly from their activation of the defence immune genes of *C. elegans*. This lifespan model of *C. elegans* can be used to screen for *C. jejuni*-antagonistic LAB on a large scale.

## Methods

### *C. elegans*, LAB and *C. jejuni*

*C. elegans* N2 Bristol wild-type strain and *E. coli* OP50 (Caenorhabditis Genetics Center, Minnepolis, University of Minnesota) was used in the study. 20 °C was used for nematodes maintence and cultivation. The procedures for the cultivation, maintenance and synchronization of the nematode have been reported [48, 55]. Before using *E. coli* OP50 on every experiment, its concentration was adjusted to 1× 10^8^ colony-forming units (CFU)/mL in sterile phosphate-buffered saline solution (PBS).

Six LAB were purchased from the American Type Culture Collection (ATCC) or the Japan Collection of Microorganisms and 38 were isolated from human faeces from different habitats and traditional fermented food. The selected LAB were identified through 16S gene sequencing. All the isolates were deposited at the Culture Collection of Food Microorganisms at Jiangnan University (Table S1). The whole genome sequence of 12 isolates, namely N8, N9, 422, 427, 430, Z5, L103, X13, JS-SZ-1-5, JS-WX-9-1, 9–5 and H27-1 L were aligned and identified as new strains of the corresponding species reported. The other 26 isolates are assumed tentative strains, for they were isolated from samples of different origin, geographical location and span over seven years. All of the LAB were cultivated in deMan, Rogosa and Sharpe (MRS) agar at a 2% (v/v) inoculum size at 37 °C for 18 to 20 h with an atmosphere of 85% N_2_, 10% CO_2_, and 5% H_2_. Before using LAB on every experiment, its concentration was adjusted to 1× 10^9^ colony-forming units (CFU)/mL in sterile phosphate-buffered saline solution (PBS).

The source and cultivation of *C. jejuni* NCTC 11168 have been reported [55]. Before using *C. jejuni* on every experiment, its concentration was adjusted to 1× 10^9^ colony-forming units (CFU)/mL in sterile phosphate-buffered saline solution (PBS).

### Life-span experimental analysis of *C. elegans*

The nematodes were synchronised as previously described [27]. To build a model for the experimental analysis of worm death induced by *C. jejuni*, 10^9^ CFU/mL of fresh and live *C. jejuni* was prepared for worms every day during the whole experiment. Each group had 80–100 worms at L4 stage and three independent replications were performed for each assay. As shown in Fig. S1, after L4 stage, worms fed only with *E. coli* OP50 formed the negative control group (OP50 group). The worms fed only with *C. jejuni* was called *C. jejuni* group. Throughout the experiment, OP 50 + *C. jejuni* group was the nematodes fed by *E. coli* OP50 and *C. jejuni* at the same time after L4 stage. In an experiment undertaken to evaluate the role of LAB in protecting the worms against death caused by *C. jejuni*, the worms were either fed 10^8^ CFU/mL *E. coli* OP50 or 10^9^ CFU/mL LAB strains for the first 3 days. After 72 h of incubation, the worms were moved to new 6 cm plates with *C. jejuni* at a concentration of 10^9^ CFU/mL. The worms that were first fed *E. coli* OP50 (72 h) and next *C. jejuni* formed the *C. jejuni* reference group (OP50*/C. jejuni* group); those treated with LAB (72 h) before *C. jejuni* were regarded as the LAB protection groups (LAB/*C. jejuni* group). A worm was considered dead when it failed to respond to gentle touch with a worm picker. The numbers of live worms were recorded, and the probability of their survival was calculated as described previously [27].

### Examination of bacterial load in the intestine of *C. elegans*

The numbers of *E. coli* OP50, *Lactobacillus* and *C. jejuni* in the nematodes’ intestine were determined with some modification of the method described previously [27]. Examination of *Lactobacillus* was executed on LAB/*C. jejuni* groups. The sampling (50 worms per sample) was done every 2 days and day 0 marked the arrival of the nematodes at the L4 stage before being fed thallus. At the same time, on day 6, *E.coli* OP50 load in all groups was detected. *C. jejuni* test also followed the same method on *Lactobacillus*. But, day 0 marked the point at which the nematodes were first fed *C. jejuni.* After surface sterilization, the worms were mashed mechanically with a pellet pestle motor, re-suspended in the M9 buffer, and inoculated onto eosin-methylene blue medium (EMB medium), MRS or Columbia blood agar for counting of *E. coli* OP50, *Lactobacillus* and *C. jejuni*, respectively. At least three independent replications were performed for each assay.

### Measurement of body size and pharynx pumping

The live worms were examined for their body size measurements every 2 days and their pharynx pumping on the eighth day after they were fed thallus at the L4 stage. Images of adult nematodes were taken with a VCT-VBIT digital microscope (Shimadzu, Kyoto, Japan) and analyzed using the ImageJ software. In this system, the area of the worm’s projection was estimated automatically and used as an index of body size. And the worm’s pharynx pumping was measured per 30s.

### Evaluation of the effects on the growth of *C. jejuni* by co-culturing *E. coli* OP50

The growth of *C. jejuni* co-cultured with *E. coli* OP50 was determined by the following method as previously described [53]. The *C. jejuni* cells (10^7^ CFU/mL) suspended in antibiotic-free brain heart infusion broth (BHIB) containing 5% serum were incubated under microaerophilic conditions for 48 h at 37 °C in the presence of a 10% volume of live *E. coli* OP50 (10^7^ CFU/mL). Both were incubated together in liquid medium. The viability of *C. jejuni* was evaluated from the number of viable CFUs in *C. jejuni* culture as described above on *C. jejuni*-selective plates. At least three independent replications were performed for this assay.

### RNA extraction, reverse transcription and quantitative real-time PCR analysis

The whole RNA of *C. elegans* and of the bacteria used in the life-span experiment was extracted. About 100 worms were prepared for the lysates. The RNA was extracted as previously described [27]. Total RNA of bacteria and *C. elegans* from the life span assays was extracted from lysates of the worms, using the mirVana miRNA Isolation Kit (Ambion, TX) according to manufacturer’s instructions.

The transcription of mitogen-activated protein kinase (MAPK) pathway genes (*tir-1*, *nsy-1*, *sek-1* and *pmk-1*), antimicrobial peptide genes (*spp-1*, *clec-85*, *abf-2*, *clec-60* and *lys-7*), Daf-16 pathway genes (*daf-16* and *age-1*), a TGF-β pathway gene (*dbl-1*) and antioxidant genes (*skn-1* and *bar-1*) in *C. elegans* was determined by quantitative polymerase chain reaction (qPCR) [49]. SuperScript first-strand synthesis system (Invitrogen, Carlsbad, CA) was used for the synthesis of cNDA. *GapA*, as a housekeeping gene, was used to determine the levels of mRNA transcription of the *C. elegans* immune genes and to normalize the input amounts of RNA. PCR primers specific to each of the genes were experimentally validated and used in the RT-qPCR assay (Table S9). One μl of each cDNA sample was included in a 24 μl reaction mixture containing 12.5 μl Master Mix, 3.75 μl each of the primers at 150 nM, and 4 μl irradiated and double autoclaved dH_2_O. The QPCR programs included 10 min at 95 °C and 40 cycles of 95 °C for 30 s, 56 °C for 1 min, and 72 °C for 30 s. Fluorescence wasmeasured after each annealing during the cycles. The delta Ct method was used to analyse the RT-qPCR data and to determine the relative abundance of the target genes (fold changes) (fold changes) [50, 51]. The data of OP50 group was taken as benchmark 1. All experiment was repeated three times.

### Induction of acute iletis for *C. jejuni* infection and LAB intervention in mice

Three-week-old female C57BL/6 mice obtained from Shanghai Laboratory Animal Center (Shanghai, China) were used in the experiments. All the experimental procedures (#JIPD2017030) have been reported [55]. All the experiments conformed to the Ministry of Science and Technology of China’s Guide for the Care and Use of Laboratory Animals.

To induce ileitis, the C57BL/6 mice were infected orally with *T. gondii* ME49 cysts according to previous reports [52, 55]. As shown in Fig. S2A, 4 days later, mice were infected with LAB and *C. jejuni* NCTC 11168 by gavage at a volume of 0.3 mL every day individually. The mice were successively treated with LAB (10^9^ CFU) and *C. jejuni* (10^9^ CFU) at 1 h intervals on 2 consecutive days. The *C. jejuni* loads in their faeces were checked on day 9. The faeces were resuspended in sterile PBS and serially diluted. The diluted samples were spread on Columbia blood agar with a *C. jejuni* selective supplement and incubated at 5% oxygen concentration at 37 °C for 48 h. After incubation, the numbers of *C. jejuni* in the samples were counted. The mice were euthanized with CO_2_ after experiment. For each mice, the treatment based on the different LAB, the blood collection and execution and analysis of indicators were all done by different investigator. The first investigator was the only person aware of the treatment group allocation. All experiment was repeated three times.

### *C. jejuni* infection and LAB intervention in chicken

White leghorn chicken eggs (Jinan Baizhun Biologic Inspection Company, Ltd., China) were maintained in an egg incubator until the chicks hatched. About 24 h after hatching, 8 chicks were randomly assigned to several groups. All the experimental procedures were approved by the Animal Care and Use Committee at Jiangsu Nannong Hi-technology company, LTD. All the experiments adhered to the Ministry of Science and Technology of China’s Guide for the Care and Use of Laboratory Animals.

As shown in Fig. S2B, all birds were administered 10^9^ CFU of *C. jejuni* NCTC 11168 in a 0.3 mL suspension by oral gavage. Twenty-four hours after oral gavage, LAB (10^9^ CFU in 0.3 mL) were orally administered daily to 11 groups (in total 88 birds) of *C. jejuni*-inoculated birds for two weeks. PBS was administered to the remaining one *C. jejuni* group of birds (8 birds). Chicks were euthanized with CO_2_ at 20% chamber replacement rate on 23 days post-inoculation, and the cecal contents were diluted in ice-cold PBS to 0.1 g/mL. Ten-fold serial dilutions of each sample were prepared and then plated on Columbia blood agar with a *C. jejuni* selective supplement and incubated at a 5% oxygen concentration at 37 °C for 48 h. For each chicken, the treatment based on the different LAB, the blood collection and execution and analysis of indicators were all done by different investigator. The first investigator was the only person aware of the treatment group allocation. All experiment was repeated three times.

### Statistical analysis

Kaplan-Meier survival analysis was used to assess the survival rate of *C. elegans*. The significance of differences on data was determined by using Student’s t-test. Means ± standard deviations (SDs) were used as a form of data pression. Differenceswere considered significant at a *P*-value of < 0.01.

## Supplementary Information

**Additional file 1 Fig. S1** Flow chart of LAB interventions in *C. elegans*. **Fig. S2** Flow chart of LAB interventions in mice and chicken. **(A)** Experimental design for mice. **(B)** Experimental design for chicken. **Table S1** The information of 44 LAB strains. **Table S2** LAB load in the intestine of *C. elegans*. **Table S3** *C. jejuni* load in the intestine of *C. elegans*. **Table S4** *E.coli* OP50 load in the intestine of *C. elegans*. **Table S5** Effects of LAB on the body size of *C. elegans* infected by *C. jejuni*. **Table S6** Effects of LAB on the pharynx pumping of *C. elegans* infected by *C. jejuni*. **Table S7** Differential effects of LAB on the transcription of immune genes of *C. elegans* infected by *C. jejuni*. **Table S8** Differential effects of LAB alone on the transcription of immune genes of *C. elegans* on day 3. **Table S9** qPCR primers for nematodes defense gene.

## Data Availability

The datasets used and/or analysed during the current study are available from the corresponding author on reasonable request.

## Abbreviations

*C. jejuni*: *Campylobacter jejuni*
LAB: lactic acid bacteria
*E. coli*: *Escherichia coli*
*C. elegans*: *Caenorhabditis elegans*
*T. gondii*: *Toxoplasma gondii*
