# Supplementary material for: Lactic acid bacteria that activate immune gene expression in Caenorhabditis elegans can antagonise Campylobacter jejuni infection in nematodes, chickens and mice
Source: BMC Microbiol. 2021 Jun 5;21:169. doi: 10.1186/s12866-021-02226-x (PMC8180125; doi:10.1186/s12866-021-02226-x)
Supplement: Supplementary file 1 — Additional file 1 Fig. S1 Flow chart of LAB interventions in C. elegans. Fig. S2 Flow chart of LAB interventions in mice and chicken. (A) Experimental design for mice. (B) Experimental design for chicken. Table S1 The information of 44 LAB strains. Table S2 LAB load in the intestine of C. elegans. Table S3 C. jejuni load in the intestine of C. elegans. Table S4 E.coli OP50 load in the intestine of C. elegans. Table S5 Effects of LAB on the body size of C. elegans infected by C. jejuni. Table S6 Effects of LAB on the pharynx pumping of C. elegans infected by C. jejuni. Table S7 Differential effects of LAB on the transcription of immune genes of C. elegans infected by C. jejuni. Table S8 Differential effects of LAB alone on the transcription of immune genes of C. elegans on day 3. Table S9 qPCR primers for nematodes defense gene. [file 12866_2021_2226_MOESM1_ESM.docx]

Supplementary Material

**Supplementary Figures and Tables**


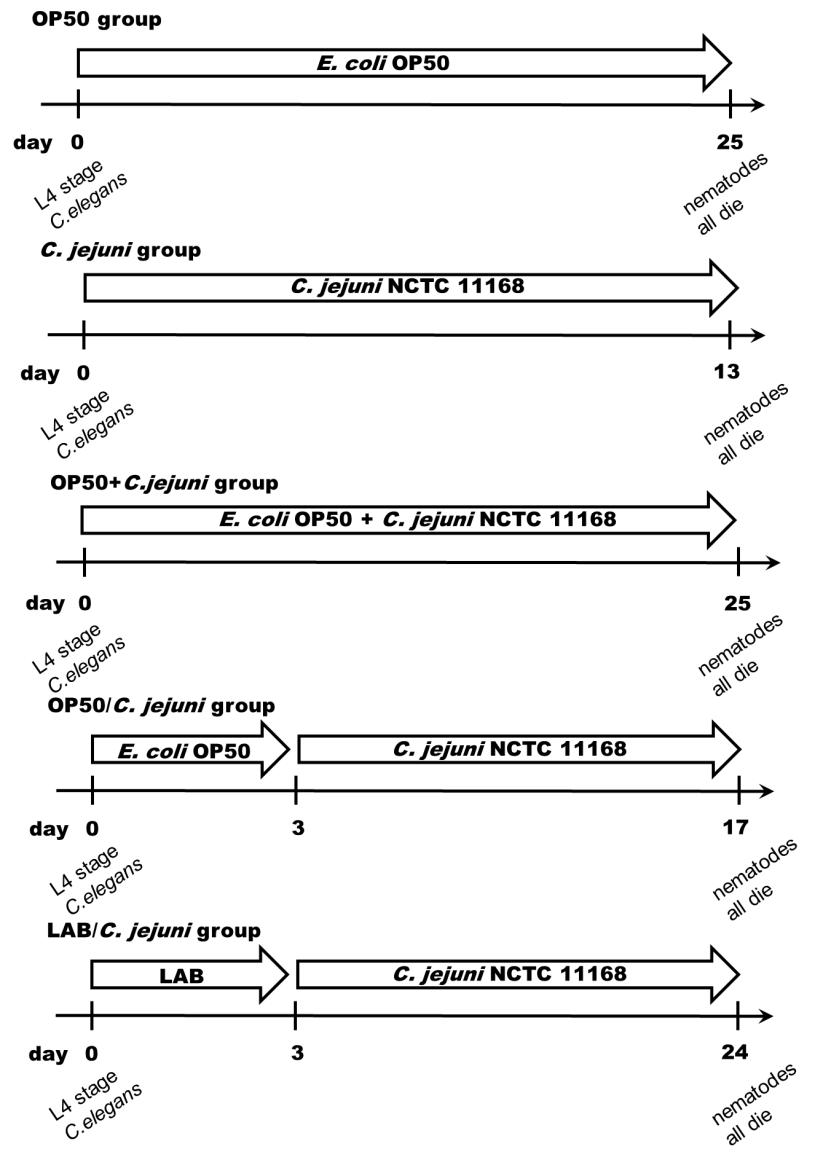


**Fig. S1** Flow chart of LAB interventions in *C. elegans*
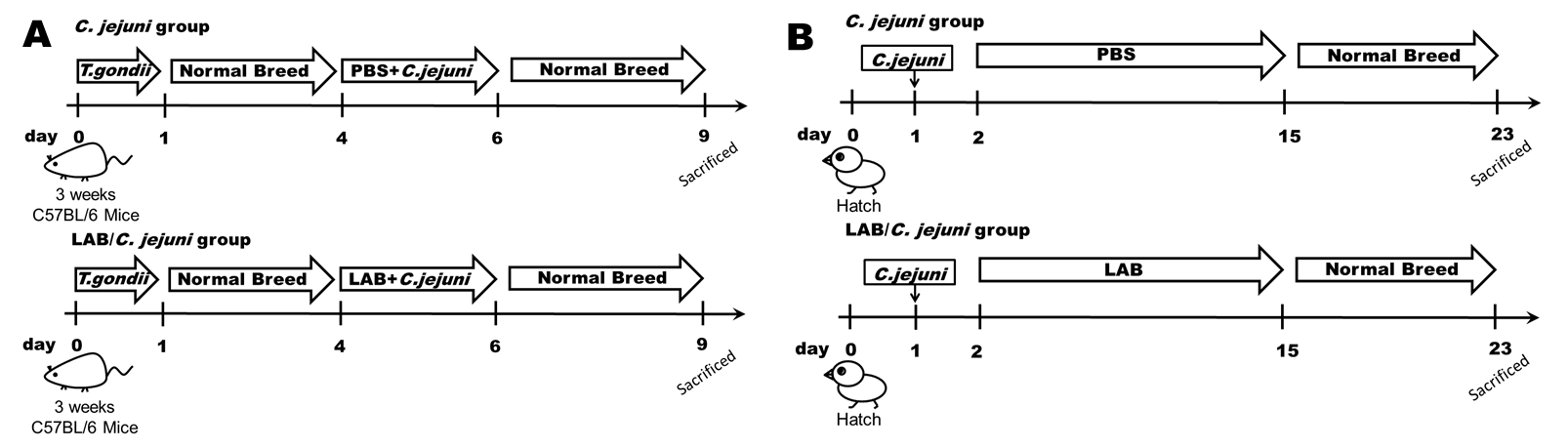
 **Fig. S2** Flow chart of LAB interventions in mice and chicken. **(A)** Experimental design for mice. **(B)** Experimental design for chicken.

**Tables**

**Table S1**

The information of 44 LAB strains

| Strain | Species | Year of isolation | Location of isolation | Origin |
| --- | --- | --- | --- | --- |
| ZX7 | *L. plantarum* | 2010 | Nantong, Jiangsu | Traditional koumiss |
| N8 | *L. plantarum* | 2010 | Panzhihua, Sichuan | Traditional koumiss |
| N9 | *L. plantarum* | 2010 | Leshan, Sichuan | Traditional koumiss |
| N34 | *L. plantarum* | 2010 | Nantong, Jiangsu | Traditional koumiss |
| 730 | *L. plantarum* | 2015 | Huhehaote,Neimengu | Traditional pickles |
| 675 | *L. plantarum* | 2015 | Bama, Guangxi | Traditional pickles |
| 408 | *L. plantarum* | 2014 | Lingao,Hainan | Traditional pickles |
| 591 | *L. plantarum* | 2015 | Meishan,Sichuan | Traditional pickles |
| 676 | *L. plantarum* | 2015 | Nantong,Jiangsu | Traditional pickles |
| PC-T7 | *L. plantarum* | 2013 | Wuxi,Jiangsu | Traditional koumiss |
| 430 | *L. plantarum* | 2014 | Lingao,Hainan | Traditional pickles |
| 427 | *L. plantarum* | 2014 | Ledong,Hainan | Traditional pickles |
| Z6 | *L. plantarum* | 2010 | Leshan, Sichuan | Traditional pickles |
| 13-7 | *L. salivarius* | 2016 | Bama, Guangxi | Healthy adult feces |
| Z5 | *L. salivarius* | 2013 | Huhehaote, Neimengu | Traditional koumiss |
| LGG | *L. rhamnosus* | - | - | ATCC 53103 |
| X14 | *L.rhamnosus* | 2010 | Nantong, Jiangsu | Traditional koumiss |
| YM-1 | *L.rhamnosus* | 2015 | Enshi,Hubei | Healthy adult feces |
| ZX6 | *L. helveticus* | 2010 | Shihezi,Xinjiang | Traditional koumiss |
| rui | *L. helveticus* | 2013 | Huhehaote,Neimengu | Healthy infant feces |
| H17 | *L. crispatus* | 2016 | Bama, Guangxi | Healthy poultry feces |
| 2009 | *L. crispatus* | - | - | JCM 2009 |
| X13 | *L. crispatus* | 2015 | Zhongxiang,Hubei | Healthy poultry feces |
| 9-5 | *L. reuteri* | 2015 | Yangzhou,Jiangsu | Healthy adult feces |
| L103 | *L. reuteri* | 2015 | Zhongxiang,Hubei | Healthy adult feces |
| G14 | *L. reuteri* | 2016 | Bama, Guangxi | Healthy adult feces |
| H9 | *L. johnsonii* | 2014 | Huhehaote,Neimengu | Healthy adult feces |
| 1101 | *L. johnsonii* | - | - | JCM 1101 |
| 13M2 | *L. johnsonii* | 2015 | Yangzhou,Jiangsu | Healthy adult feces |
| 11657 | *L. gasseri* | - | - | JCM 11657 |
| LSQ3 | *L. gasseri* | 2015 | Yangzhou,Jiangsu | Healthy adult feces |
| JS-SZ-1-5 | *L. gasseri* | 2016 | Wuxi, Jiangsu | Healthy infant feces |
| JS-WX-9-1 | *L. gasseri* | 2016 | Wuxi, Jiangsu | Healthy infant feces |
| N29 | *L. fermentum* | 2010 | Leshan, Sichuan | Traditional koumiss |
| Z7 | *L. fermentum* | 2010 | Panzhihua, Sichuan | Traditional koumiss |
| 422 | *L. fermentum* | 2010 | Leshan, Sichuan | Traditional pickles |
| B | *L. fermentum* | 2010 | Wuxi, Jiangsu | Healthy infant feces |
| G20 | *L. gallinarum* | 2013 | Wuxi, Jiangsu | Healthy poultry feces |
| 1132 | *L. acidophilus* | - | - | JCM 1132 |
| 720 | *L. acidophilus* | 2015 | Zhongxiang,Hubei | Healthy adult feces |
| NCFM | *L. acidophilus* | - | - | ATCC SD5221 |
| H33M-1 | *P. pentosaceus* | 2017 | Zhongxiang,Hubei | Healthy poultry feces |
| H29M-8M | *P. pentosaceus* | 2017 | Zhongxiang,Hubei | Healthy poultry feces |
| H27-1L | *P. pentosaceus* | 2015 | Zhongxiang,Hubei | Healthy poultry feces |

**Table S2**

LAB load in the intestine of *C. elegans*

| Groups^a^ | 2^nd^ day(log CFU/worm) | 4^th^ day(log CFU/worm) | 6^th^ day(log CFU/worm) | Groups | 2^nd^ day(log CFU/worm) | 4^th^ day(log CFU/worm) | 6^th^ day(log CFU/worm) |
| --- | --- | --- | --- | --- | --- | --- | --- |
| 422/*C. jejuni* | 4.01±0.04 | 4.53±0.01 | 4.51±0.03 | PC-T7/*C. jejuni* | 3.97±0.01 | 4.21±0.03 | 4.11±0.02 |
| B/*C. jejuni* | 3.91±0.06 | 3.87±0.13 | 4.01±0.07 | JS-WX-9-1/*C. jejuni* | 4.28±0.03 | 4.31±0.07 | 4.33±0.12 |
| G14/*C. jejuni* | 3.78±0.04 | 3.94±0.18 | 3.97±0.09 | N8/*C. jejuni* | 5.01±0.12* | 4.97±0.15* | 4.95±0.06* |
| X14/*C. jejuni* | 4.21±0.05 | 4.54±0.07* | 4.47±0.13 | 11657/*C. jejuni* | 3.86±0.04 | 3.87±0.06 | 3.74±0.04 |
| LSQ3/*C. jejuni* | 4.01±0.11 | 4.03±0.07 | 4.05±0.02 | YM-1/*C. jejuni* | 4.56±0.11* | 4.32±0.0.07 | 4.47±0.01 |
| 591/*C. jejuni* | 4.87±0.14* | 4.85±0.02* | 4.91±0.15* | 676/*C. jejuni* | 4.51±0.04 | 4.53±0.18* | 4.61±0.19* |
| H17/*C. jejuni* | 4.14±0.06 | 4.08±0.12 | 4.21±0.08 | JS-SZ-1-5/*C. jejuni* | 4.61±0.02* | 4.57±0.08* | 4.68±0.07* |
| LGG/*C. jejuni* | 4.53±0.11* | 4.57±0.13* | 4.71±0.12* | NCFM/*C. jejuni* | 4.84±0.14* | 4.86±0.05* | 4.71±0.04* |
| 1101/*C. jejuni* | 4.36±0.15 | 4.41±0.06 | 4.37±0.01 | N34/*C. jejuni* | 5.03±0.08* | 5.21±0.15* | 5.19±0.03* |
| N29/*C. jejuni* | 3.71±0.06 | 3.87±0.02 | 3.89±0.03 | 427/*C. jejuni* | 4.53±0.07* | 5.01±0.12* | 5.07±0.13* |
| 408/*C. jejuni* | 4.53±0.03* | 4.71±0.05* | 4.72±0.01* | X13/*C. jejuni* | 5.01±0.03* | 5.21±0.01* | 5.17±0.03* |
| H29M-8M/*C. jejuni* | 3.51±0.12 | 3.68±0.04 | 3.74±0.09 | 720/*C. jejuni* | 4.65±0.14* | 4.67±0.03* | 4.74±0.08* |
| 730/*C. jejuni* | 5.01±0.11* | 4.81±0.05* | 4.82±0.03* | 2009/*C. jejuni* | 4.86±0.11* | 4.69±0.01* | 4.81±0.05* |
| H9/*C. jejuni* | 3.98±0.07 | 4.05±0.10 | 4.11±0.12 | H33M-1/*C. jejuni* | 3.98±0.07 | 4.01±0.08 | 4.11±0.12 |
| ZX7/*C. jejuni* | 3.51±0.07 | 3.71±0.05 | 3.65±0.15 | 13M2/*C. jejuni* | 4.69±0.08* | 4.58±0.12* | 4.59±0.09* |
| 430/*C. jejuni* | 4.71±0.16* | 4.83±0.08* | 4.78±0.04* | L103/*C. jejuni* | 5.11±0.14* | 5.27±0.06* | 5.14±0.16* |
| 675/*C. jejuni* | 4.63±0.08* | 4.62±0.03* | 4.65±0.02* | G20/*C. jejuni* | 5.01±0.05* | 4.78±0.11* | 4.95±0.04* |
| Z7/*C. jejuni* | 3.81±0.11 | 3.79±0.08 | 3.82±0.05 | 1132/*C. jejuni* | 4.56±0.05* | 4.62±0.14* | 4.63±0.07* |
| Z6/*C. jejuni* | 4.51±0.02* | 4.49±0.08 | 4.53±0.07* | H27-1L/*C. jejuni* | 4.86±0.07* | 4.95±0.04* | 4.76±0.05* |
| rui/*C. jejuni* | 4.01±0.04 | 3.98±0.02 | 3.96±0.01 | 13-7/*C. jejuni* | 4.58±0.06* | 4.63±0.11* | 4.79±0.02* |
| 9-5/*C. jejuni* | 4.51±0.02* | 4.63±0.03* | 4.61±0.02* | Z5/*C. jejuni* | 5.21±0.03* | 5.17±0.01* | 5.31±0.04* |
| ZX6/*C. jejuni* | 4.73±0.02* | 4.78±0.06* | 4.75±0.02* | N9/*C. jejuni* | 4.50±0.07 | 4.41±0.03 | 4.57±0.12* |

^a^ Day 0 marked the arrival of the nematodes at the L4 stage before being fed thallus. The graphs show means ± SDs. * Indicates statistically significant differences at *p* < 0.05.

**Table S3**

*C. jejuni* load in the intestine of *C. elegans*.

| Groups^a^ | 2^nd^ day(log CFU/worm) | 4^th^ day(log CFU/worm) | 6^th^ day(log CFU/worm) | Groups | 2^nd^ day(log CFU/worm) | 4^th^ day(log CFU/worm) | 6^th^ day(log CFU/worm) |
| --- | --- | --- | --- | --- | --- | --- | --- |
| OP50/*C. jejuni* | 3.73±0.11 | 4.15±0.07 | 4.54±0.09 | PC-T7/*C. jejuni* | 3.63±0.01 | 3.97±0.02 | 3.95±0.08 |
| 422/*C. jejuni* | 3.78±0.11 | 4.17±0.06 | 4.68±0.02 | JS-WX-9-1/*C. jejuni* | 3.58±0.05 | 4.08±0.10 | 4.23±0.03 |
| B/*C. jejuni* | 3.71±0.03 | 4.13±0.02 | 4.71±0.07 | N8/*C. jejuni* | 3.53±0.01 | 3.87±0.08 | 3.86±0.02* |
| G14/*C. jejuni* | 3.64±0.13 | 3.94±0.05 | 3.91±0.11 | 11657/*C. jejuni* | 3.67±0.07 | 3.96±0.02 | 3.99±0.07 |
| X14/*C. jejuni* | 3.58±0.12 | 3.81±0.03 | 3.92±0.01 | YM-1/*C. jejuni* | 3.52±0.12 | 3.67±0.08* | 3.71±0.07* |
| LSQ3/*C. jejuni* | 3.68±0.07 | 4.04±0.14 | 4.48±0.06 | 676/*C. jejuni* | 3.62±0.05 | 3.69±0.13* | 3.65±0.04** |
| 591/*C. jejuni* | 3.51±0.02 | 3.59±0.02* | 3.52±0.07** | JS-SZ-1-5/*C. jejuni* | 3.54±0.07 | 3.64±0.01* | 3.57±0.20** |
| H17/*C. jejuni* | 3.71±0.08 | 3.85±0.12 | 3.94±0.05 | NCFM/*C. jejuni* | 3.62±0.01 | 3.67±0.07* | 3.64±0.09** |
| LGG/*C. jejuni* | 3.62±0.03 | 4.15±0.14 | 4.57±0.13 | N34/*C. jejuni* | 3.62±0.04 | 3.67±0.05* | 3.59±0.07** |
| 1101/*C. jejuni* | 3.71±0.06 | 3.64±0.08* | 3.84±0.07* | 427/*C. jejuni* | 3.51±0.07 | 3.57±0.17* | 3.48±0.08** |
| N29/*C. jejuni* | 3.62±0.09 | 3.85±0.14 | 4.01±0.12 | X13/*C. jejuni* | 3.61±0.11 | 3.62±0.04* | 3.51±0.02** |
| 408/*C. jejuni* | 3.61±0.06 | 3.61±0.15* | 3.55±0.11** | 720/*C. jejuni* | 3.51±0.07 | 3.76±0.05 | 3.96±0.05 |
| H29M-8M/*C. jejuni* | 3.71±0.11 | 3.96±0.07 | 4.54±0.09 | 2009/*C. jejuni* | 3.64±0.09 | 3.63±0.12* | 3.51±0.05** |
| 730/*C. jejuni* | 3.53±0.08 | 3.73±0.01 | 3.69±0.08** | H33M-1/*C. jejuni* | 3.57±0.02 | 3.57±0.11* | 3.43±0.03** |
| H9/*C. jejuni* | 3.61±0.07 | 4.08±0.06 | 4.47±0.09 | 13M2/*C. jejuni* | 3.58±0.04 | 3.62±0.05* | 3.54±0.14** |
| ZX7/*C. jejuni* | 3.51±0.03 | 3.92±0.01 | 3.85±0.04* | L103/*C. jejuni* | 3.62±0.11 | 3.56±0.04* | 3.56±0.19** |
| 430/*C. jejuni* | 3.63±0.12 | 3.71±0.11 | 3.67±0.04* | G20/*C. jejuni* | 3.61±0.06 | 3.63±0.08* | 3.59±0.05** |
| 675/*C. jejuni* | 3.63±0.05 | 3.73±0.11 | 3.72±0.03* | 1132/*C. jejuni* | 3.59±0.06 | 3.51±0.03* | 3.64±0.07** |
| Z7/*C. jejuni* | 3.63±0.07 | 4.01±0.06 | 3.87±0.02* | H27-1L/*C. jejuni* | 3.23±0.05 | 3.54±0.03* | 3.48±0.04** |
| Z6/*C. jejuni* | 3.72±0.02 | 4.21±0.01 | 4.63±0.05 | 13-7/*C. jejuni* | 3.52±0.03 | 3.53±0.12* | 3.63±0.14** |
| rui/*C. jejuni* | 3.53±0.05 | 3.72±0.01 | 3.71±0.11* | Z5/*C. jejuni* | 3.51±0.11 | 3.48±0.13* | 3.45±0.04** |
| 9-5/*C. jejuni* | 3.61±0.15 | 3.79±0.03 | 3.82±0.07* | N9/*C. jejuni* | 3.57±0.04 | 3.61±0.11* | 3.60±0.08** |
| ZX6/*C. jejuni* | 3.53±0.01 | 3.64±0.17* | 3.65±0.08** |  |  |  |  |

^a^ Day 0 marked the point at which the nematodes were first fed *C. jejuni*. The graphs show means ± SDs. * Indicates statistically significant differences at *p* < 0.05. ** Indicates statistically significant differences at *p* < 0.01.

**Table S4**

*E.coli* OP50 load in the intestine of *C. elegans*.

| Groups^a^ | 6^th^ day(log CFU/worm) | Groups | 6^th^ day(log CFU/worm) | Groups | 6^th^ day(log CFU/worm) | Groups | 6^th^ day(log CFU/worm) |
| --- | --- | --- | --- | --- | --- | --- | --- |
| OP50/*C. jejuni* | 1.53±0.08 | H29M-8M/*C. jejuni* | 1.47±0.06 | PC-T7/*C. jejuni* | 1.49±0.03 | 720/*C. jejuni* | 1.21±0.08 |
| 422/*C. jejuni* | 1.32±0.12 | 730/*C. jejuni* | 1.52±0.04 | JS-WX-9-1/*C. jejuni* | 1.33±0.07 | 2009/*C. jejuni* | 1.43±0.07 |
| B/*C. jejuni* | 1.24±0.03 | H9/*C. jejuni* | 1.36±0.08 | N8/*C. jejuni* | 1.54±0.06 | H33M-1/*C. jejuni* | 1.64±0.14 |
| G14/*C. jejuni* | 1.52±0.04 | ZX7/*C. jejuni* | 1.36±0.04 | 11657/*C. jejuni* | 1.42±0.04 | 13M2/*C. jejuni* | 1.32±0.07 |
| X14/*C. jejuni* | 1.51±0.09 | 430/*C. jejuni* | 1.46±0.05 | YM-1/*C. jejuni* | 1.27±0.16 | L103/*C. jejuni* | 1.25±0.11 |
| LSQ3/*C. jejuni* | 1.48±0.11 | 675/*C. jejuni* | 1.48±0.03 | 676/*C. jejuni* | 1.41±0.11 | G20/*C. jejuni* | 1.54±0.09 |
| 591/*C. jejuni* | 1.61±0.09 | Z7/*C. jejuni* | 1.61±0.02 | JS-SZ-1-5/*C. jejuni* | 1.60±0.09 | 1132/*C. jejuni* | 1.43±0.06 |
| H17/*C. jejuni* | 1.53±0.08 | Z6/*C. jejuni* | 1.39±0.05 | NCFM/*C. jejuni* | 1.37±0.17 | H27-1L/*C. jejuni* | 1.37±0.0 |
| LGG/*C. jejuni* | 1.56±0.03 | rui/*C. jejuni* | 1.47±0.02 | N34/*C. jejuni* | 1.39±0.05 | 13-7/*C. jejuni* | 1.34±0.14 |
| 1101/*C. jejuni* | 1.48±0.14 | 9-5/*C. jejuni* | 1.37±0.10 | 427/*C. jejuni* | 1.36±0.03 | Z5/*C. jejuni* | 1.43±0.11 |
| N29/*C. jejuni* | 1.52±0.02 | ZX6/*C. jejuni* | 1.29±0.08 | X13/*C. jejuni* | 1.49±0.11 | N9/*C. jejuni* | 1.51±0.02 |
| 408/*C. jejuni* | 1.49±0.01 |  |  |  |  |  |  |

^a^ Day 0 marked the arrival of the nematodes at the L4 stage before being fed thallus. The graphs show means ± SDs. * Indicates statistically significant differences at *p* < 0.05.

**Table S5**

Effects of LAB on the body size of *C. elegans* infected by *C. jejuni*

| Groups^a^ | 4^th^ day(mm^2^) | 6^th^ day(mm^2^) | 8^th^ dya(mm^2^) | Groups | 4^th^ day(mm^2^) | 6^th^ day(mm^2^) | 8^th^ dya(mm^2^) |
| --- | --- | --- | --- | --- | --- | --- | --- |
| OP50/*C. jejuni* | 0.0951±0.0019 | 0.113±0.0075 | 0.115 ±0.0101 | PC-T7/*C. jejuni* | 0.0961±0.0042 | 0.0983±0.0021 | 0.107±0.0017 |
| 422/*C. jejuni* | 0.0963±0.0039 | 0.112±0.0074 | 0.116±0.0017 | JS-WX-9-1/*C. jejuni* | 0.0931±0.0019 | 0.0978±0.0047 | 0.1077±0.0134 |
| B/*C. jejuni* | 0.0951±0.0079 | 0.0974±0.0083 | 0.0999±0.0094 | N8/*C. jejuni* | 0.0936±0.0075 | 0.0991±0.0071 | 0.0999±0.0113 |
| G14/*C. jejuni* | 0.0971±0.0021 | 0.0993±0.0087 | 0.107±0.0138 | 11657/*C. jejuni* | 0.0971±0.0054 | 0.0931±0.0076 | 0.0964±0.0118 |
| X14/*C. jejuni* | 0.0963±0.0037 | 0.101±0.0039 | 0.111±0.0171 | YM-1/*C. jejuni* | 0.0936±0.0068 | 0.0982±0.0082 | 0.0994±0.0054 |
| LSQ3/*C. jejuni* | 0.0954±0.0014 | 0.1047±0.0047 | 0.08942±0.0119 | 676/*C. jejuni* | 0.0621±0.0031* | 0.0718±0.0065* | 0.0827±0.0101* |
| 591/*C. jejuni* | 0.0903±0.0043 | 0.0978±0.0011 | 0.117±0.0122 | JS-SZ-1-5/*C. jejuni* | 0.0995±0.0077 | 0.0956±0.0053 | 0.0937±0.0028 |
| H17/*C. jejuni* | 0.0947±0.0022 | 0.1104±0.0016 | 0.1021±0.0111 | NCFM/*C. jejuni* | 0.0912±0.0046 | 0.0987±0.0014 | 0.1084±0.0132 |
| LGG/*C. jejuni* | 0.0899±0.0017 | 0.0953±0.0053 | 0.103±0.0109 | N34/*C. jejuni* | 0.0593±0.0023* | 0.0613±0.0015* | 0.0699±0.0072* |
| 1101/*C. jejuni* | 0.0984±0.0054 | 0.0991±0.0024 | 0.0897±0.0016 | 427/*C. jejuni* | 0.0589±0.0057* | 0.0634±0.0083* | 0.0701±0.0123* |
| N29/*C. jejuni* | 0.0943±0.0071 | 0.101±0.0111 | 0.111±0.0178 | X13/*C. jejuni* | 0.0927±0.0051 | 0.102±0.0047 | 0.113±0.0099 |
| 408/*C. jejuni* | 0.0951±0.0053 | 0.0973±0.0013 | 0.107±0.0123 | 720/*C. jejuni* | 0.0933±0.0014 | 0.0971±0.0016 | 0.0899±0.0104 |
| H29M-8M/*C. jejuni* | 0.0931±0.0064 | 0.0974±0.1001 | 0.0934±0.0071 | 2009/*C. jejuni* | 0.0953±0.0072 | 0.0955±0.0038 | 0.0986±0.0045 |
| 730/*C. jejuni* | 0.0928±0.0047 | 0.0975±0.0011 | 0.103±0.0021 | H33M-1/*C. jejuni* | 0.0984±0.0058 | 0.0964±0.0131 | 0.0941±0.0087 |
| H9/*C. jejuni* | 0.0985±0.0078 | 0.0987±0.0016 | 0.1056±0.0047 | 13M2/*C. jejuni* | 0.0967±0.0017 | 0.1188±0.0049 | 0.0931±0.0011 |
| ZX7/*C. jejuni* | 0.0956±0.0047 | 0.101±0.0081 | 0.117±0.0099 | L103/*C. jejuni* | 0.0944±0.135 | 0.0941±0.0027 | 0.0966±0.0104 |
| 430/*C. jejuni* | 0.0972±0.0034 | 0.0973±0.0042 | 0.105±0.0107 | G20/*C. jejuni* | 0.0913±0.0027 | 0.1164±0.0117 | 0.1213±0.0125 |
| 675/*C. jejuni* | 0.0593±0.0036* | 0.0678±0.0071* | 0.0753±0.0105* | 1132/*C. jejuni* | 0.0984±0.0044 | 0.0951±0.0033 | 0.1117±0.0066 |
| Z7/*C. jejuni* | 0.0899±0.0021 | 0.0971±0.0038 | 0.108±0.078 | H27-1L/*C. jejuni* | 0.0911±0.0101 | 0.1102±0.0047 | 0.0978±0.0015 |
| Z6/*C. jejuni* | 0.0936±0.0015 | 0.102±0.0078 | 0.105±0.0053 | 13-7/*C. jejuni* | 0.0912±0.0007 | 0.0998±0.0038 | 0.112±0.0071 |
| rui/*C. jejuni* | 0.0938±0.0051 | 0.0961±0.0058 | 0.108±0.0111 | Z5/*C. jejuni* | 0.975±0.0071 | 0.115±0.0061 | 0.121±0.0098 |
| 9-5/*C. jejuni* | 0.1087±0.0017 | 0.0937±00.0031 | 0.0970±0.0018 | N9/*C. jejuni* | 0.091±0.0078 | 0.0957±0.0011 | 0.1031±0.021 |
| ZX6/*C. jejuni* | 0.0973±0.0051 | 0.105±0.0073 | 0.117±0.0181 |  |  |  |  |

^a^ Day 0 marked the arrival of the nematodes at the L4 stage before being fed thallus. The graphs show means ± SDs. Column labelled with different superscript letters (a, b,c) showed significant differences (*p*<0.05).

**Table S6**

Effects of LAB on the pharynx pumping of *C. elegans* infected by *C. jejuni*

| Groups^a^ | Pharynx pumping (per 30s) | Groups | Pharynx pumping (per 30s) | Groups | Pharynx pumping (per 30s) | Groups | Pharynx pumping (per 30s) |
| --- | --- | --- | --- | --- | --- | --- | --- |
| OP50/*C. jejuni* | 55.4±2.1 | H29M-8M/*C. jejuni* | 51.7±2.4 | PC-T7/*C. jejuni* | 55.4±2.2 | 720/*C. jejuni* | 54.7±1.1 |
| 422/*C. jejuni* | 55.7±1.7 | 730/*C. jejuni* | 55.9±1.3 | JS-WX-9-1/*C. jejuni* | 51.7±3.1 | 2009/*C. jejuni* | 56.4±3.1 |
| B/*C. jejuni* | 52.4±1.1 | H9/*C. jejuni* | 49.7±3.1 | N8/*C. jejuni* | 53.4±2.9 | H33M-1/*C. jejuni* | 53.8±2.1 |
| G14/*C. jejuni* | 57.5±1.5 | ZX7/*C. jejuni* | 56.6±1.8 | 11657/*C. jejuni* | 49.6±3.5 | 13M2/*C. jejuni* | 56.6±1.9 |
| X14/*C. jejuni* | 53.3±0.5 | 430/*C. jejuni* | 50.5±1.3 | YM-1/*C. jejuni* | 51.2±0.7 | L103/*C. jejuni* | 53.8±2.9 |
| LSQ3/*C. jejuni* | 55.4±2.1 | 675/*C. jejuni* | 30.9±1.1* | 676/*C. jejuni* | 33±1.6* | G20/*C. jejuni* | 51.4±1.5 |
| 591/*C. jejuni* | 53.9±1.5 | Z7/*C. jejuni* | 57.7±1.9 | JS-SZ-1-5/*C. jejuni* | 56.6±0.4 | 1132/*C. jejuni* | 55.9±3.7 |
| H17/*C. jejuni* | 52.9±1.7 | Z6/*C. jejuni* | 51.4±2.9 | NCFM/*C. jejuni* | 53.4±2.8 | H27-1L/*C. jejuni* | 49.6±3.3 |
| LGG/*C. jejuni* | 51.5±0.5 | rui/*C. jejuni* | 52.7±1.5 | N34/*C. jejuni* | 36±1.5* | 13-7/*C. jejuni* | 53.5±0.5 |
| 1101/*C. jejuni* | 57.3±0.7 | 9-5/*C. jejuni* | 48.6±3.8 | 427/*C. jejuni* | 32±2.1* | Z5/*C. jejuni* | 56.1±2.4 |
| N29/*C. jejuni* | 56.8±2.4 | ZX6/*C. jejuni* | 51.7±1.5 | X13/*C. jejuni* | 54.6±1.5 | N9/*C. jejuni* | 51.5±1.5 |
| 408/*C. jejuni* | 54.2±2.1 |  |  |  |  |  |  |

^a^ Pharynx pumping (per 30s) of *C. elegans* treated with LAB and *C. jejuni* on day 8. Day 0 marked the arrival of the nematodes at the L4 stage before being fed thallus. The graphs show means ± SDs. Column labelled with different superscript letters (a, b) showed significant differences (*p*<0.05). Any two columns with same superscript letter.

**Table S7**

Differential effects of LAB on the transcription of immune genes of *C. elegans* infected by *C. jejuni*

| Groups^a^ | Fold Change | | | | | | | | | | | | | |
| --- | --- | --- | --- | --- | --- | --- | --- | --- | --- | --- | --- | --- | --- | --- |
|  | *tir-1* | *nsy-1* | *sek-1* | *pmk-1* | *spp-1* | *clec-85* | *abf-2* | *clec-60* | *lys-7* | *daf-16* | *age-1* | *dbl-1* | *skn-1* | *bar-1* |
| OP50 | 1±0.03 | 1±0.11 | 1±0.05 | 1±0.04 | 1±0.01 | 1±0.03 | 1±0.10 | 1±0.07 | 1±0.01 | 1±0.02 | 1±0.02 | 1±0.03 | 1±0.01 | 1±0.06 |
| OP50/*C. jejuni* | 2.42±0.18 | 0.69±0.10 | 0.72±0.01 | 1.32±0.15 | 0.92±0.04 | 0.81±0.03 | 0.24±0.02 | 0.20±0.04 | 0.16±0.01 | 1.11±0.01 | 1.12±0.09 | 1.35±0.16 | 1.20±0.07 | 2.55±0.05 |
| 422/*C. jejuni* | 1.02±0.15 | 0.82±0.06 | 0.39±0.03 | 0.57±0.03 | 0.37±0.02 | 0.51±0.01 | 0.13±0.02 | 0.22±0.07 | 0.11±0.01 | 0.77±0.05 | 0.59±0.05 | 1.18±0.11 | 1.69±0.03 | 0.80±0.01 |
| B/*C. jejuni* | 3.93±0.05 | 0.11±0.01 | 0.94±0.06 | 1.03±0.01 | 0.42±0.03 | 1.80±0.04 | 0.21±0.01 | 0.37±0.03 | 0.70±0.06 | 0.64±0.02 | 0.06±0.01 | 1.64±0.12 | 4.02±0.22 | 1.00±0.01 |
| G14/*C. jejuni* | 1.92±0.13 | 0.73±0.02 | 0.56±0.02 | 0.43±0.04 | 0.26±0.01 | 0.36±0.01 | 0.12±0.01 | 0.70±0.03 | 0.34±0.01 | 0.67±0.01 | 0.92±0.01 | 1.34±0.15 | 1.82±0.01 | 1.10±0.02 |
| X14/*C. jejuni* | 2.48±0.15 | 0.86±0.04 | 0.72±0.02 | 1.41±0.01 | 1.13±0.01 | 0.75±0.03 | 0.25±0.01 | 0.62±0.09 | 0.12±0.01 | 0.91±0.01 | 1.45±0.02 | 1.02±0.08 | 1.26±0.04 | 0.94±0.05 |
| LSQ3/*C. jejuni* | 1.23±0.02 | 0.74±0.01 | 0.36±0.02 | 0.54±0.01 | 0.56±0.01 | 0.57±0.02 | 0.13±0.02 | 0.25±0.01 | 1.25±0.01 | 2.04±0.02 | 0.08±0.01 | 1.03±0.01 | 0.84±0.02 | 1.04±0.01 |
| 591/*C. jejuni* | 2.12±0.01 | 0.61±0.01 | 0.47±0.01 | 1.21±0.03 | 1.02±0.03 | 0.69±0.02 | 0.29±0.03 | 0.36±0.04 | 0.55±0.02 | 0.88±001 | 0.12±0.01 | 1.26±0.08 | 0.96±0.01 | 1.08±0.07 |
| H17/*C. jejuni* | 0.96±0.05 | 0.94±0.02 | 0.89±0.02 | 0.96±0.01 | 1.56±0.01 | 0.84±0.0.3 | 1.13±0.01 | 0.58±0.01 | 0.34±0.01 | 0.17±0.02 | 1.06±0.02 | 1.36±0.01 | 1.56±0.08 | 0.69±0.03 |
| LGG/*C. jejuni* | 1.24±0.03 | 1.25±0.04 | 0.16±0.01 | 1.17±0.05 | 0.57±0.02 | 1.25±0.01 | 0.56±0.02 | 1.52±0.03 | 1.58±0.01 | 0.51±0.02 | 2.01±0.03 | 1.04±0.04 | 2.46±0.07 | 1.41±0.01 |
| 1101/*C. jejuni* | 0.84±0.02 | 1.41±0.02 | 0.82±0.03 | 1.64±0.08 | 0.63±0.01 | 1.08±0.0.2 | 0.68±0.04 | 0.25±0.01 | 1.69±0.02 | 0.56±0.02 | 1.25±0.02 | 0.58±0.02 | 0.96±0.02 | 0.73±0.06 |
| N29/*C. jejuni* | 1.46±0.06 | 0.67±0.03 | 0.93±0.01 | 0.65±0.03 | 0.85±0.01 | 0.86±0.01 | 1.69±0.02 | 0.84±0.01 | 0.58±0.01 | 1.52±0.01 | 0.59±0.01 | 1.25±0.01 | 0.58±0.01 | 0.69±0.01 |
| 408/*C. jejuni* | 1.23±0.01 | 0.84±0.01 | 0.46±0.03 | 2.01±0.06 | 0.56±0.02 | 1.23±0.04 | 0.27±0.03 | 0.97±0.02 | 0.11±0.02 | 0.83±0.01 | 1.25±0.01 | 1.36±0.02 | 1.69±0.07 | 1.08±0.03 |
| H29M-8M/*C. jejuni* | 1.58±0.01 | 0.87±0.02 | 0.85±0.01 | 0.68±0.04 | 1.05±0.03 | 0.96±0.06 | 1.06±0.01 | 0.88±0.02 | 0.51±0.01 | 0.32±0.04 | 0.31±0.02 | 1.18±0.03 | 0.96±0.02 | 1.37±0.02 |
| 730/*C. jejuni* | 1.26±0.03 | 0.69±0.04 | 0.32±0.02 | 0.69±0.01 | 1.26±0.04 | 0.25±0.01 | 1.21±0.01 | 0.41±0.01 | 2.68±0.01 | 2.56±0.02 | 0.69±0.02 | 1.31±0.01 | 3.64±0.04 | 1.08±0.03 |
| H9/*C. jejuni* | 0.85±0.01 | 1.21±0.04 | 1.12±0.08 | 0.84±0.02 | 1.17±0.01 | 0.34±0.0.3 | 0.36±.02 | 0.46±0.02 | 3.58±0.01 | 1.18±0.01 | 0.47±0.01 | 1.07±0.01 | 2.05±0.01 | 1.16±0.06 |
| ZX7/*C. jejuni* | 1.46±0.03 | 1.34±0.04 | 0.34±0.01 | 0.46±0.01 | 1.36±0.01 | 0.28±0.01 | 0.58±0.01 | 0.82±0.04 | 0.79±0.02 | 0.49±0.01 | 1.11±0.02 | 1.08±0.02 | 0.77±0.02 | 1.01±0.02 |
| 430/*C. jejuni* | 2.99±0.07 | 1.05±0.01 | 0.97±0.10 | 1.53±0.03 | 1.07±0.02 | 2.66±0.14 | 0.44±0.01 | 2.76±0.03 | 0.19±0.01 | 1.72±0.02 | 1.85±0.03 | 1.51±0.20 | 1.01±0.28 | 2.76±0.09 |
| 675/*C. jejuni* | 2.15±0.04 | 1.01±0.01 | 0.42±0.02 | 2.59±0.01 | 0.58±0.02 | 1.86±0.03 | 0.75±0.02 | 0.85±0.01 | 0.22±0.02 | 1.44±0.01 | 2.13±0.01 | 0.9±0.14 | 1.09±0.08 | 2.01±0.03 |
| Z7/*C. jejuni* | 2.56±0.01 | 1.08±0.02 | 0.86±0.0.3 | 1.64±0.05 | 0.64±0.03 | 2.09±0.03 | 1.52±0.03 | 1.58±0.01 | 1.23±0.05 | 0.21±0.01 | 0.51±0.01 | 0.69±0.08 | 1.36±0.07 | 2.01±0.03 |
| Z6/*C. jejuni* | 1.61±0.09 | 1.02±0.05 | 0.59±0.01 | 0.98±0.01 | 0.80±0.02 | 0.68±0.01 | 0.22±0.02 | 0.68±0.04 | 0.24±0.02 | 1.04±0.05 | 0.93±0.06 | 1.41±0.08 | 2.12±0.14 | 1.62±0.05 |
| rui/*C. jejuni* | 1.48±0.03 | 0.84±0.01 | 0.75±0.02 | 0.84±0.02 | 0.91±0.02 | 0.84±0.02 | 0.16±0.01 | 0.74±0.02 | 2.58±0.01 | 1.36±0.05 | 0.12±0.01 | 1.26±0.17 | 1.28±0.07 | 0.58±0.02 |
| 9-5/*C. jejuni* | 2.48±0.04 | 1.23±0.07 | 0.93±0.04 | 0.63±0.04 | 0.56±0.01 | 1.36±0.09 | 0.47±0.02 | 2.11±0.01 | 2.61±0.02 | 0.85±0.02 | 0.09±0.01 | 1.58±0.06 | 2.58±0.03 | 0.36±0.01 |
| ZX6/*C. jejuni* | 3.12±0.04 | 0.85±0.02 | 1.23±0.04 | 1.87±0.05 | 0.76±0.03 | 2.41±0.01 | 0.69±0.02 | 1.47±0.03 | 0.58±0.01 | 2.14±0.01 | 0.84±0.02 | 1.36±0.02 | 1.02±0.01 | 2.58±0.14 |
| PC-T7/*C. jejuni* | 3.16±0.02 | 0.80±0.01 | 0.78±0.04 | 1.28±0.01 | 0.46±0.01 | 0.67±0.03 | 0.44±0.01 | 1.05±0.06 | 0.46±0.03 | 1.19±0.02 | 1.39±0.02 | 1.63±0.06 | 2.72±0.41 | 1.52±0.18 |
| JS-WX-9-1/*C. jejuni* | 2.46±0.02 | 0.69±0.02 | 1.21±0.01 | 2.12±0.04 | 2.43±0.02 | 0.88±0.01 | 1.02±0.04 | 1.66±0.02 | 1.09±0.02 | 1.80±0.01 | 2.31±0.01 | 1.02±0.01 | 1.36±0.05 | 1.36±0.08 |
| N8/*C. jejuni* | 3.45±0.06 | 1.17±0.03 | 0.57±0.02 | 0.84±0.02 | 0.97±0.02 | 0.69±0.03 | 1.29±0.01 | 1.58±0.02 | 2.04±0.03 | 2.56±0.01 | 1.06±0.01 | 0.74±0.03 | 2.05±0.01 | 0.58±0.02 |
| 11657/*C. jejuni* | 2.14±0.02 | 0.69±0.01 | 0.69±0.04 | 0.96±.01 | 1.25±0.03 | 1.84±0.0.1 | 0.71±0.02 | 0.71±0.01 | 1.57±0.01 | 2.41±0.01 | 0.08±0.01 | 0.36±0.01 | 0.69±0.04 | 1.36±0.03 |
| YM-1/*C. jejuni* | 1.42±0.03 | 1.36±0.01 | 0.71±0.08 | 2.01±0.01 | 2.34±.03 | 1.02±0.04 | 0.69±0.03 | 1.07±0.02 | 0.69±0.01 | 0.44±0.01 | 1.17±0.01 | 1.25±0.02 | 0.21±0.01 | 0.36±0.01 |
| 676/*C. jejuni* | 3.14±0.06 | 0.58±0.04 | 1.11±0.01 | 2.36±0.02 | 0.57±0.01 | 1.25±0.01 | 0.77±0.01 | 1.36±0.01 | 0.44±.01 | 1.84±0.03 | 0.69±0.02 | 1.36±0.03 | 1.06±0.01 | 2.07±0.06 |
| JS-SZ-1-5/*C. jejuni* | 3.25±0.03 | 1.11±0.05 | 0.46±0.02 | 1.86±0.02 | 2.56±0.04 | 0.85±0.02 | 1.51±0.0.2 | 1.25±0.01 | 3.58±0.01 | 1.86±0.01 | 0.58±0.01 | 2.51±0.01 | 2.59±0.04 | 1.05±0.09 |
| NCFM/*C. jejuni* | 3.36±0.07 | 0.78±0.04 | 1.56±0.02 | 1.63±0.02 | 0.84±0.02 | 2.56±0.02 | 1.58±0.01 | 0.72±0.02 | 2.48±0.02 | 0.69±0.03 | 0.36±0.02 | 0.47±0.01 | 3.61±0.09 | 0.36±0.04 |
| N34/*C. jejuni* | 3.39±0.11 | 1.23±0.01 | 1.86±0.02 | 2.51±0.07 | 0.99±0.05 | 1.54±0.01 | 1.36±0.02 | 0.81±0.01 | 2.11±0.01 | 2.47±0.02 | 1.58±0.01 | 2.31±0.03 | 0.87±0.01 | 2.48±0.16 |
| 427/*C. jejuni* | 2.99±0.07 | 0.87±0.06 | 0.92±0.05 | 1.76±0.03 | 2.33±0.01 | 0.97±0.06 | 1.08±0.08 | 0.36±0.01 | 0.59±0.01 | 1.88±0.05 | 1.78±0.06 | 1.41±0.06 | 2.10±0.10 | 2.78±0.18 |
| X13/*C. jejuni* | 1.78±0.07 | 0.73±0.04 | 0.57±0.06 | 1.68±0.04 | 3.41±0.04 | 0.85±0.02 | 1.84±0.02 | 0.25±0.02 | 3.45±0.02 | 3.64±0.01 | 1.25±0.01 | 1.07±0.02 | 3.84±0.05 | 2.06±0.02 |
| 720/*C. jejuni* | 3.45±0.01 | 0.86±0.02 | 0.74±0.01 | 0.96±0.04 | 2.14±0.0.2 | 0.96±0.04 | 0.57±0.01 | 0.46±0.02 | 4.99±0.02 | 3.14±0.03 | 2.24±0.01 | 1.11±0.17 | 3.11±0.09 | 1.43±0.06 |
| 2009/*C. jejuni* | 3.74±0.04 | 0.94±0.03 | 1.84±0.06 | 2.61±0.07 | 2.36±0.04 | 2.13±0.01 | 2.51±0.06 | 0.33±0.01 | 2.44±0.02 | 0.85±0.01 | 1.69±0.03 | 2.22±0.01 | 0.69±0.03 | 1.25±0.02 |
| H33M-1/*C. jejuni* | 2.64±0.06 | 1.18±0.07 | 1.64±0.01 | 2.16±0.08 | 2.58±0.0.4 | 2.56±0.08 | 0.94±0.01 | 2.16±0.04 | 3.58±0.01 | 0.69±0.02 | 2.05±0.01 | 1.58±0.01 | 3.84±0.09 | 0.85±0.04 |
| 13M2/*C. jejuni* | 2.91±0.07 | 1.31±0.01 | 1.55±0.02 | 1.41±0.07 | 3.46±0.01 | 3.01±0.02 | 2.71±0.03 | 0.55±0.02 | 4.15±0.02 | 1.56±0.02 | 0.69±0.01 | 1.98±0.05 | 4.21±0.16 | 2.28±0.03 |
| L1103/*C. jejuni* | 2.46±0.03 | 1.93±0.01 | 1.84±0.01 | 2.77±0.12 | 4.12±0.01 | 3.56±0.04 | 2.97±0.01 | 0.67±0.01 | 3.58±0.02 | 2.16±0.01 | 2.10±0.01 | 2.51±0.21 | 4.08±0.06 | 2.46±0.05 |
| G20/*C. jejuni* | 2.47±0.07 | 1.22±0.02 | 2.76±0.02 | 1.99±0.06 | 1.54±0.01 | 1.61±0.02 | 0.26±0.01 | 0.52±0.01 | 0.39±0.01 | 1.73±0.04 | 0.81±0.04 | 1.94±0.02 | 1.44±0.10 | 2.12±0.46 |
| 1132/*C. jejuni* | 3.17±0.03 | 1.76±0.02 | 2.54±0.08 | 2.45±0.03 | 3.85±0.04 | 1.84±0.01 | 2.14±0.02 | 1.68±0.03 | 3.69±0.05 | 3.19±0.01 | 1.85±0.06 | 1.55±0.02 | 3.96±0.08 | 2.61±0.09 |
| H27-1L/*C. jejuni* | 2.16±0.05 | 1.68±0.01 | 2.98±0.01 | 1.85±0.01 | 4.01±.05 | 2.94±0.04 | 2.78±0.05 | 2.56±0.01 | 4.15±0.02 | 2.69±0.01 | 2.37±0.06 | 1.51±0.06 | 2.41±0.03 | 1.36±0.02 |
| 13-7/*C. jejuni* | 3.02±0.23 | 1.23±0.17 | 1.45±0.16 | 1.69±0.02 | 4.92±0.01 | 1.75±0.07 | 0.53±0.01 | 2.26±0.01 | 4.34±0.01 | 3.82±0.01 | 1.91±0.11 | 1.17±0.04 | 4.75±0.14 | 2.49±0.06 |
| Z5/*C. jejuni* | 3.94±0.06 | 1.64±0.01 | 0.65±0.05 | 1.37±0.02 | 4.40±0.01 | 2.58±0.15 | 2.37±0.01 | 1.39±0.05 | 4.25±0.02 | 1.64±0.02 | 2.36±0.07 | 1.45±0.09 | 1.72±0.01 | 2.53±0.11 |
| N9/*C. jejuni* | 3.75±0.11 | 1.83±0.01 | 1.91±0.04 | 2.65±0.02 | 3.99±0.01 | 2.69±0.12 | 2.45±0.01 | 0.28±0.01 | 4.49±0.01 | 2.52±0.01 | 1.41±0.18 | 2.05±0.01 | 4.16±0.01 | 2.35±0.02 |

^a^ Day 0 marked the arrival of the nematodes at the L4 stage before being fed thallus.

**Table S8**

Differential effects of LAB alone on the transcription of immune genes of *C. elegans* on day 3

| Groups^a^ | Fold Change | | | | | | | | | | | | | |
| --- | --- | --- | --- | --- | --- | --- | --- | --- | --- | --- | --- | --- | --- | --- |
|  | *tir-1* | *nsy-1* | *sek-1* | *pmk-1* | *spp-1* | *clec-85* | *abf-2* | *clec-60* | *lys-7* | *daf-16* | *age-1* | *dbl-1* | *skn-1* | *bar-1* |
| OP50 | 1±0.02 | 1±0.11 | 1±0.05 | 1±0.04 | 1±0.01 | 1±0.03 | 1±0.10 | 1±0.07 | 1±0.01 | 1±0.02 | 1±0.02 | 1±0.03 | 1±0.01 | 1±0.06 |
| 422 | 3.59±0.36* | 1.28±0.34 | 1.23±0.41 | 1.08±0.18 | 0.90±0.18 | 0.93±0.11 | 1.04±0.29 | 0.84±0.12 | 1.29±0.25 | 0.82±0.11 | 1.36±0.29 | 0.92±0.27 | 1.39±0.34 | 0.84±0.31 |
| B | 0.96±0.17 | 1.69±0.15 | 0.87±0.42 | 0.91±0.28 | 1.09±0.36 | 1.38±0.14 | 0.84±0.15 | 1.45±0.21 | 1.09±0.10 | 1.56±0.17 | 0.89±28 | 0.79±0.21 | 1.28±0.19 | 1.21±0.36 |
| G14 | 1.25±0.46 | 0.87±0.24 | 0.91±0.24 | 1.23±0.39 | 0.86±0.23 | 0.87±0.36 | 0.81±0.39 | 0.90±0.33 | 1.09±0.34 | 1.13±0.41 | 0.99±0.18 | 1.56±0.13 | 0.91±0.25 | 0.96±0.27 |
| X14 | 0.84±0.02 | 1.09±0.03 | 1.21±0.03 | 1.21±0.01 | 1.14±0.11 | 1.05±0.03 | 1.22±0.04 | 0.87±0.02 | 0.95±0.01 | 0.84±002 | 1.02±001 | 1.13±0.03 | 0.88±0.02 | 1.02±0.04 |
| LSQ3 | 0.83±0.01 | 0.91±0.05 | 1.09±0.12 | 1.13±0.04 | 1.15±0.05 | 1.01±0.12 | 0.92±0.03 | 0.94±0.03 | 1.16±0.02 | 1.29±0.02 | 1.29±0.03 | 1.05±0.01 | 1.01±0.01 | 0.84±0.02 |
| 591 | 1.22±0.01 | 1.13±0.01 | 1.04±0.13 | 0.96±0.02 | 0.91±0.06 | 0.84±0.02 | 1.19±0.05 | 0.89±0.01 | 0.88±0.03 | 0.83±0.03 | 1.29±0.01 | 0.81±0.03 | 1.03±0.02 | 1.21±0.01 |
| H17 | 1.21±0.01 | 0.94±0.01 | 1.21±0.06 | 1.18±0.04 | 1.27±0.03 | 1.22±0.03 | 0.99±0.02 | 0.83±0.01 | 1.05±0.01 | 1.23±0.01 | 1.21±0.05 | 0.84±0.01 | 1.29±0.03 | 0.94±0.01 |
| LGG | 1.17±0.12 | 0.95±0.03 | 1.04±0.07 | 0.99±0.03 | 1.06±0.04 | 1.29±0.04 | 1.18±0.01 | 1.09±0.02 | 1.13±0.02 | 1.11±0.02 | 0.85±0.06 | 1.17±0.01 | 1.04±0.04 | 1.06±0.03 |
| 1101 | 0.96±0.04 | 0.98±0.05 | 1.34±0.01 | 1.32±0.04 | 1.28±0.01 | 1.25±0.02 | 1.17±0.01 | 1.35±0.03 | 1.25±0.03 | 0.92±0.05 | 1.24±0.01 | 1.24±0.06 | 1.18±0.07 | 1.41±0.04 |
| N29 | 1.36±0.03 | 1.42±0.04 | 1.26±0.01 | 1.27±0.01 | 0.85±0.02 | 1.26±0.03 | 1.04±0.02 | 1.21±0.05 | 1.04±0.01 | 1.37±0.01 | 0.87±0.04 | 1.32±0.03 | 1.22±0.06 | 1.13±0.03 |
| 408 | 1.12±0.02 | 1.09±0.11 | 1.43±0.02 | 0.83±0.01 | 0.86±0.01 | 1.23±0.04 | 1.44±0.04 | 1.11±0.06 | 1.28±0.02 | 1.21±0.06 | 1.22±0.07 | 1.53±0.02 | 1.04±0.02 | 0.92±0.05 |
| H29M-8M | 1.01±0.01 | 1.42±0.03 | 1.36±0.03 | 0.83±0.03 | 1.44±0.01 | 0.84±0.01 | 1.13±0.03 | 1.35±0.03 | 1.44±0.06 | 1.16±0.04 | 0.83±0.03 | 1.31±0.02 | 1.06±0.01 | 1.25±0.01 |
| 730 | 1.25±0.01 | 1.13±0.04 | 1.45±0.03 | 1.25±0.02 | 1.43±0.03 | 0.87±0.02 | 1.03±0.01 | 1.4±0.02 | 1.45±0.01 | 1.11±0.01 | 0.95±0.09 | 0.89±.05 | 1.23±0.01 | 1.23±0.04 |
| H9 | 1.32±0.03 | 1.23±0.02 | 1.37±0.01 | 0.98±0.02 | 1.27±0.04 | 1.41±0.02 | 1.26±0.02 | 1.23±0.05 | 0.98±0.02 | 1.16±0.03 | 1.59±0.01 | 1.08±0.01 | 1.25±0.02 | 1.06±0.03 |
| ZX7 | 1.3±0.03 | 0.97±0.01 | 1.14±0.03 | 1.31±0.03 | 1.15±0.02 | 1.14±0.01 | 0.87±0.04 | 1.02±0.01 | 1.23±0.03 | 1.11±0.04 | 1.47±0.05 | 1.22±0.03 | 1.47±0.03 | 1.43±0.01 |
| 430 | 1.47±0.14 | 0.78±0.38 | 0.87±0.03 | 0.79±0.18 | 0.93±0.19 | 1.32±0.45 | 1.36±0.41 | 1.06±0.33 | 0.99±0.22 | 0.93±0.41 | 0.93±0.13 | 1.01±0.38 | 0.77±0.34 | 1.27±0.31 |
| 675 | 1.06±0.02 | 1.39±0.03 | 1.48±0.01 | 1.46±0.01 | 1.33±0.02 | 0.93±0.02 | 1.29±0.04 | 1.48±0.01 | 1.18±0.01 | 1.13±0.03 | 1.27±0.01 | 1.39±0.06 | 1.34±0.02 | 0.99±0.02 |
| Z7 | 0.98±0.01 | 0.96±0.05 | 0.62±0.12 | 1.21±0.04 | 0.67±0.01 | 0.99±0.01 | 1.22±0.03 | 1.13±0.03 | 0.99± | 1.18±0.04 | 0.63±0.03 | 1.17±0.02 | 0.72±0.01 | 1.13±0.03 |
| Z6 | 1.05±0.11 | 0.86±0.17 | 0.96±0.19 | 1.09±0.21 | 1.24±0.17 | 0.87±0.41 | 0.88±0. 11 | 0.87±0.69 | 1.39±0.18 | 0.84±0.34 | 1.11±0.28 | 1.39±0.48 | 1.27±0.42 | 1.29±0.55 |
| rui | 0.71±0.01 | 1.19±0.04 | 0.75±0.04 | 0.93± | 0.87±0.03 | 1.25±0.03 | 1.24±0.04 | 1.16±0.04 | 1.05±0.03 | 0.8±0.01 | 0.87±0.06 | 0.92±0.01 | 1.07±0.01 | 0.94±0.05 |
| 9-5 | 0.87±0.01 | 0.77±0.03 | 0.69±0.03 | 1.25±0.03 | 0.89±0.02 | 0.98±0.02 | 1.22±0.03 | 1.04±0.02 | 0.65±0.02 | 1.15±0.03 | 0.91±0.03 | 1.16±0.03 | 1.25±0.02 | 0.81±0.01 |
| ZX6 | 1.18±0.06 | 1.26±0.01 | 1.04±0.06 | 1.28±0.02 | 0.72±0.03 | 1.19±0.01 | 1.09±0.06 | 1.08±0.11 | 0.63±0.01 | 0.88±0.07 | 0.65±0.02 | 1.25±0.02 | 0.68±0.01 | 0.74±0.02 |
| PC-T7 | 0.84±0.32 | 0.91±0.41 | 1.36±0.28 | 1.36±0.22 | 0.99±0.38 | 1.07±0.16 | 0.93±0.24 | 1.39±0.22 | 1.09±0.21 | 0.89±0. 49 | 0.97±0.41 | 1.04±0.17 | 0.79±0.32 | 1.17±0.61 |
| JS-WX91 | 0.66±0.04 | 1.13±0.01 | 1.09±0.01 | 1.24±0.01 | 1.04±0.04 | 1.01±0.02 | 0.76±0.01 | 1.03±0.01 | 1.04±0.04 | 1.28±0.08 | 1.02±0.04 | 0.84±0.01 | 1.13±0.01 | 0.76±0.01 |
| N8 | 0.97±0.03 | 0.96±0.01 | 0.78±0.02 | 0.92±0.02 | 1.29±0.02 | 1.09±0.01 | 0.79±0.03 | 1.13±0.03 | 1.09±0.03 | 0.81±0.02 | 0.74±0.11 | 1.29±0.02 | 0.63±0.03 | 0.95±0.02 |
| 11657 | 0.92±0.03 | 0.89±0.02 | 0.81±0.03 | 1.01±0.04 | 0.78±0.03 | 0.81±0.03 | 0.91±0.01 | 1.06±0.02 | 0.95±0.01 | 0.73±0.03 | 0.95±0.06 | 1.13±0.0.3 | 0.97±0.02 | 1.25±0.03 |
| YM-1 | 0.71±0.01 | 0.96±0.02 | 1.22±0.01 | 0.99±0.01 | 1.32±0.01 | 0.82±0.04 | 0.95±0.04 | 0.84±0.05 | 0.99±0.04 | 1.29±0.04 | 1.21±0.07 | 1.23±0.04 | 0.83±0.01 | 1.04±0.01 |
| 676 | 0.98±0.02 | 0.82±0.03 | 1.09±0.11 | 1.25±0.03 | 0.71±0.07 | 1.22±0.02 | 1.25±0.03 | 0.97±0.06 | 0.75±0.02 | 1.04±0.01 | 0.62±0.09 | 0.78±0.03 | 0.74±0.03 | 0.91±0.02 |
| JS-SZ15 | 0.77±0.03 | 1.08±0.04 | 0.96±0.01 | 1.14±0.01 | 1.01±0.01 | 1.01±0.01 | 0.84±0.01 | 0.87±0.02 | 1.36±0.12 | 1.11±0.03 | 1.03±0.04 | 0.97±0.02 | 1.27±0.01 | 0.73±0.03 |
| NCFM | 0.86±0.04 | 1.23±0.03 | 0.75±0.02 | 1.11±0.02 | 1.07±0.03 | 1.33±0.01 | 0.81±0.02 | 0.72±0.02 | 0.68±0.05 | 0.82±0.02 | 0.62±0.01 | 0.62±0.01 | 1.05±0.03 | 1.15±0.04 |
| N34 | 1.29±0.05 | 1.08±0.02 | 0.92±0.03 | 1.28±0.03 | 1.15±0.01 | 0.74±0.02 | 0.85±0.03 | 0.64±0.01 | 1.23±0.04 | 1.02±0.04 | 1.18±0.03 | 1.09±0.01 | 0.61±0.02 | 0.87±0.02 |
| 427 | 0.85±0.46 | 1.03±0.41 | 1.03±0.29 | 0.97±0.14 | 1.17±0.47 | 1.11±0.16 | 1.13±0.37 | 0.96±0.37 | 0.96±0.43 | 1.48±0.22 | 1.37±0.51 | 1.04±0.25 | 0.82±0.36 | 1.05±0.17 |
| X13 | 0.67±0.01 | 0.94±0.01 | 1.15±0.05 | 1.25±0.01 | 1.06±0.02 | 0.74±0.03 | 0.98±0.02 | 1.21±0.03 | 1.17±0.01 | 0.63±0.02 | 1.23±0.01 | 1.29±0.01 | 1.09±0.02 | 1.02±0.03 |
| 720 | 0.66±0.03 | 1.25±0.03 | 1.22±0.06 | 1.19±0.02 | 0.89±0.01 | 0.64±0.05 | 1.02±0.01 | 1.11±0.06 | 0.72±0.04 | 0.95±0.01 | 0.68±0.02 | 0.61±0.02 | 0.89±0.03 | 1.19±0.01 |
| 2009 | 0.91±0.05 | 0.98±0.02 | 0.91±0.04 | 1.12±0.01 | 1.05±0.02 | 0.84±0.04 | 0.74±0.01 | 0.67±0.04 | 0.66±0.02 | 0.65±0.03 | 1.03±0.03 | 1.17±0.03 | 0.84±0.01 | 1.21±0.02 |
| H33M-1 | 1.12±0.02 | 1.09±0.01 | 1.13±0.01 | 1.13±0.01 | 0.92±0.03 | 1.09±0.03 | 0.66±0.01 | 1.22±0.08 | 0.68±0.03 | 1.27±0.04 | 1.34±0.06 | 1.19±0.04 | 0.64±0.02 | 0.99±0.01 |
| 13M2 | 1.16±0.04 | 0.83±0.03 | 1.03±0.02 | 1.22±0.05 | 1.26±0.01 | 0.81±0.01 | 1.23±0.02 | 1.29±0.12 | 0.99±0.02 | 0.63±0.01 | 1.17±0.04 | 0.73±0.02 | 0.63±0.01 | 0.72±0.01 |
| L103 | 1.07±0.03 | 0.89±0.01 | 0.73±0.02 | 1.03±0.03 | 0.75±0.02 | 1.12±0.06 | 0.85±0.03 | 1.21±0.13 | 1.21±0.04 | 0.87±0.05 | 0.93±0.04 | 1.12±0.01 | 1.21±0.03 | 0.94±0.03 |
| G20 | 0.91±0.21 | 1.20±0.37 | 1.09±0.43 | 1.05±0.46 | 0.81±0.39 | 1.36±0.21 | 0.96±0.36 | 0.83±0.42 | 1.31±0.24 | 1.36±0.13 | 1.56±0.12 | 0.96±0.33 | 1.09±0.24 | 1.08±0.37 |
| 1132 | 0.93±0.0 | 0.84±0.02 | 0.83±0.03 | 0.78±0.02 | 1.14±0.04 | 1.27±0.06 | 0.69±0.03 | 1.32±0.02 | 0.97±0.01 | 0.71±0.03 | 1.15±0.01 | 1.26±0.01 | 1.15±0.02 | 0.87±0.05 |
| H27-1L | 0.94±0.11 | 1.06±0.01 | 1.15±0.05 | 0.93±0.01 | 1.21±0.01 | 0.92±0.04 | 1.29±0.01 | 1.09±0.03 | 1.26±0.02 | 1.29±0.01 | 0.74±0.01 | 0.62±0.03 | 1.06±0.03 | 1.11±0.01 |
| 13-7 | 1.12±0.13 | 0.95±0.29 | 1.02±0.13 | 1.04±0.11 | 0.98±0.26 | 1.21±0.17 | 1.06±0.14 | 0.89±0.18 | 1.04±0.31 | 1.21±0.06 | 1.03±0.15 | 0.86±0.09 | 0.84±0.35 | 1.34±0.09 |
| Z5 | 0.93±0.27 | 0.96±0.21 | 1.42±0.22 | 1.36±0.24 | 1.46±0.31 | 1.23±0.40 | 0.91±0.25 | 0.94±0.14 | 1.46±0.27 | 1.03±0.25 | 0.93±0.41 | 0.86±0.09 | 1.08±0.12 | 1.28±0.33 |
| N9 | 1.28±0.42 | 3.45±0.56 | 1.06±0.31 | 0.96±0.42 | 1.06±0.15 | 0.86±0.41 | 0.97±0.28 | 0.79±0.56 | 1.23±0.13 | 1.03±0.47 | 1.36±0.24 | 1.28±0.14 | 0.96±0.41 | 1.31±0.28 |

^a^ Day 0 marked the arrival of the nematodes at the L4 stage before being fed thallus.

* Indicates statistically significant differences at *p* < 0.05.

**Table S9**

qPCR primers for nematodes defense gene

| Gene | Primer sequence（5’- 3’） | Bp |
| --- | --- | --- |
| *act-1* | F: 5’-CCCCACTCAATCCAAAGGCT-3’  R: 5’-GTACGTCCGGAAGCGTAGAG-3’ | 121 |
| *daf-16* | F: 5’-TCGTCTCGTGTTTCTCCAGC-3’  R: 5’-TAATCGGCTTCGACTCCTGC-3’ | 181 |
| *age-1* | F: 5’-CTCCTGAACCGACTGCCAAT-3’ | 359 |
|  | R: 5’-AAATGCGAGTTCGGAGAGCA-3’ |  |
| *lys-7* | F: 5’-GTACAGCGGTGGAGTCACTG-3’ | 153 bp |
|  | R: 5’-GCCTTGAGCACATTTCCAGC-3’ |  |
| *clec-60* | F: 5’-CGGTTTCAATGCGGTATGGC-3’ | 219 |
|  | R: 5’-TGAAGCTGTGGTTGAGGCAT-3’ |  |
| *clec-85* | F: 5’-CCAATGGGATGACGGAACCA-3’  R: 5’-CTTCTGTCCAGCCAACGTCT-3’ | 121 |
| *abf-3* | F: 5’-AACAGATTGGGGTCAGCTCG-3’  R: 5’-TGGAGACCATTATTGCCGGG-3’ | 189 |
| *spp-1* | F: 5’-TGGACTATGCTGTTGCCGTT-3’ | 106 |
|  | R: 5’-ACGCCTTGTCTGGAGAATCC-3’ |  |
| *abf-2* | F: 5’-CCGTTCCCTTTTCCTTGCAC-3’ | 176 |
|  | R: 5’-GACGACCGCTTCGTTTCTTG-3’ |  |
| *tir-1* | F: 5’-TTGGGTGCACAAAGAGCTGA-3’  R: 5’-GGTCGGTGTCGTTCTGTTCA-3’ | 223 |
| *nsy-1* | F: 5’-AGCGGCTCGATCAACAAGAA-3’  R: 5’-CCCATTCCACCGATATGCGA-3’ | 122 |
| *sek-1* | F: 5’-CACTGTTTGGCGACGATGAG-3’ | 158 |
|  | R: 5’-ATTCCGTCCACGTTGCTGAT-3’ |  |
| *pmk-1* | F: 5’-CCAAAAATGACTCGCCGTGA-3’ | 115 |
|  | R: 5’-CCAAAAATGACTCGCCGTGA-3’ |  |
| *bar-1* | F: 5’-CATGGTAGTCCGCGACTTGT-3’ | 119 bp |
|  | R: 5’-CGAGAATTGACCAGCTCCAGA-3’ |  |
| *skn-1* | F: 5’-CTGGCATCCTCTACCACCAC-3’ | 153 |
|  | R: 5’-TTGGTGATGATGGCCGTGTT-3’ |  |
| *dbl-1* | F: 5’- TTTTGCGGCGAACAAATCGT-3’ | 194 |
|  | R: 5’-TTCGCTGTTGCCTGTTTGTG-3’ |  |
